# Gene Diversification of an Emerging Pathogen: A Decade of Mutation in a Novel Fish Viral Hemorrhagic Septicemia (VHS) Substrain since Its First Appearance in the Laurentian Great Lakes

**DOI:** 10.1371/journal.pone.0135146

**Published:** 2015-08-27

**Authors:** Carol A. Stepien, Lindsey R. Pierce, Douglas W. Leaman, Megan D. Niner, Brian S. Shepherd

**Affiliations:** 1 Great Lakes Genetics/Genomics Laboratory, Lake Erie Center and Department of Environmental Sciences, The University of Toledo, Toledo, Ohio, 43616, United States of America; 2 Department of Biological Sciences, The University of Toledo, Toledo, Ohio, 43606, United States of America; 3 ARS/USDA/University of Wisconsin at Milwaukee/School of Freshwater Sciences, Milwaukee, Wisconsin, 53204, United States of America; National Cheng Kung University, TAIWAN

## Abstract

Viral Hemorrhagic Septicemia virus (VHSv) is an RNA rhabdovirus, which causes one of the world's most serious fish diseases, infecting >80 freshwater and marine species across the Northern Hemisphere. A new, novel, and especially virulent substrain—VHSv-IVb—first appeared in the Laurentian Great Lakes about a decade ago, resulting in massive fish kills. It rapidly spread and has genetically diversified. This study analyzes temporal and spatial mutational patterns of VHSv-IVb across the Great Lakes for the novel non-virion (*Nv*) gene that is unique to this group of novirhabdoviruses, in relation to its glycoprotein (*G*), phosphoprotein (*P*), and matrix (*M*) genes. Results show that the *Nv-*gene has been evolving the fastest (*k* = 2.0x10^-3^ substitutions/site/year), with the *G*-gene at ~1/7 that rate (*k* = 2.8x10^-4^). Most (all but one) of the 12 unique *Nv-* haplotypes identified encode different amino acids, totaling 26 changes. Among the 12 corresponding *G-*gene haplotypes, seven vary in amino acids with eight total changes. The *P-* and *M-* genes are more evolutionarily conserved, evolving at just ~1/15 (*k* = 1.2x10^-4^) of the *Nv*-gene’s rate. The 12 isolates contained four *P*-gene haplotypes with two amino acid changes, and six *M*-gene haplotypes with three amino acid differences. Patterns of evolutionary changes coincided among the genes for some of the isolates, but appeared independent in others. New viral variants were discovered following the large 2006 outbreak; such differentiation may have been in response to fish populations developing resistance, meriting further investigation. Two 2012 variants were isolated by us from central Lake Erie fish that lacked classic VHSv symptoms, having genetically distinctive *Nv*-, *G*-, and *M*-gene sequences (with one of them also differing in its *P*-gene); they differ from each other by a *G*-gene amino acid change and also differ from all other isolates by a shared *Nv*-gene amino acid change. Such rapid evolutionary differentiation may allow new viral variants to evade fish host recognition and immune responses, facilitating long-time persistence along with expansion to new geographic areas.

## Introduction

The evolutionary trajectories of RNA viruses frequently are characterized by rapid mutational changes (up to one nucleotide substitution per replication), due to their small genomes, lack of polymerase proofreading, and short generation times [1]. For example, a study by Pierce and Stepien [2] described extensive sequence diversification for Viral Hemorrhagic Septicemia virus (VHSv), which is a single-stranded RNA rhabdovirus that has infected and killed >80 fish species across the Northern Hemisphere [3]. Prior analyses of the virus’ phylogenetic and biogeographic patterns revealed that the endemic European strains—VHSv I, II, and III—comprise a clade that is the sister group to strain IV in North America [2]. The latter (as substrain IVa) first appeared along the coast of the Pacific Northwest in 1988, killing salmonids and other ecologically and economically important species [4]. Over a decade ago, a novel, new, and especially virulent substrain—IVb—appeared in the North American Laurentian Great Lakes, whose first occurrence was traced back to a sample from Lake St. Clair in 2003 [5]. Large outbreaks of VHSv-IVb occurred throughout many areas of the Great Lakes in 2006, killing >30 native fish species [6,7]. Substrain IVb is genetically and geographically distinct from all other types of VHSv, including substrain IVa [2]; however, relatively little is known of its evolutionary diversification patterns, forming the basis for the present study.

The objective of our investigation is to analyze the relative evolutionary and biogeographic differentiation patterns of VHSv-IVb among different genes over temporal and spatial distribution in the Great Lakes, building on some prior knowledge of its glycoprotein (*G*-) gene variability [2,6]. Studies have indicated that some VHSv-IVb *G*-gene variants differ in their relative virulence [8]. Swift differentiation of genetic variants may allow the virus to evade host immune responses, persist in populations, and spread to new areas and hosts [2,9]. The present study thus evaluates mutational changes in VHSv-IVb across different genes, spanning the decade following its discovery and emergence in the Great Lakes, in order to elucidate common and/or unique evolutionary diversification patterns.

**Fig 1:**
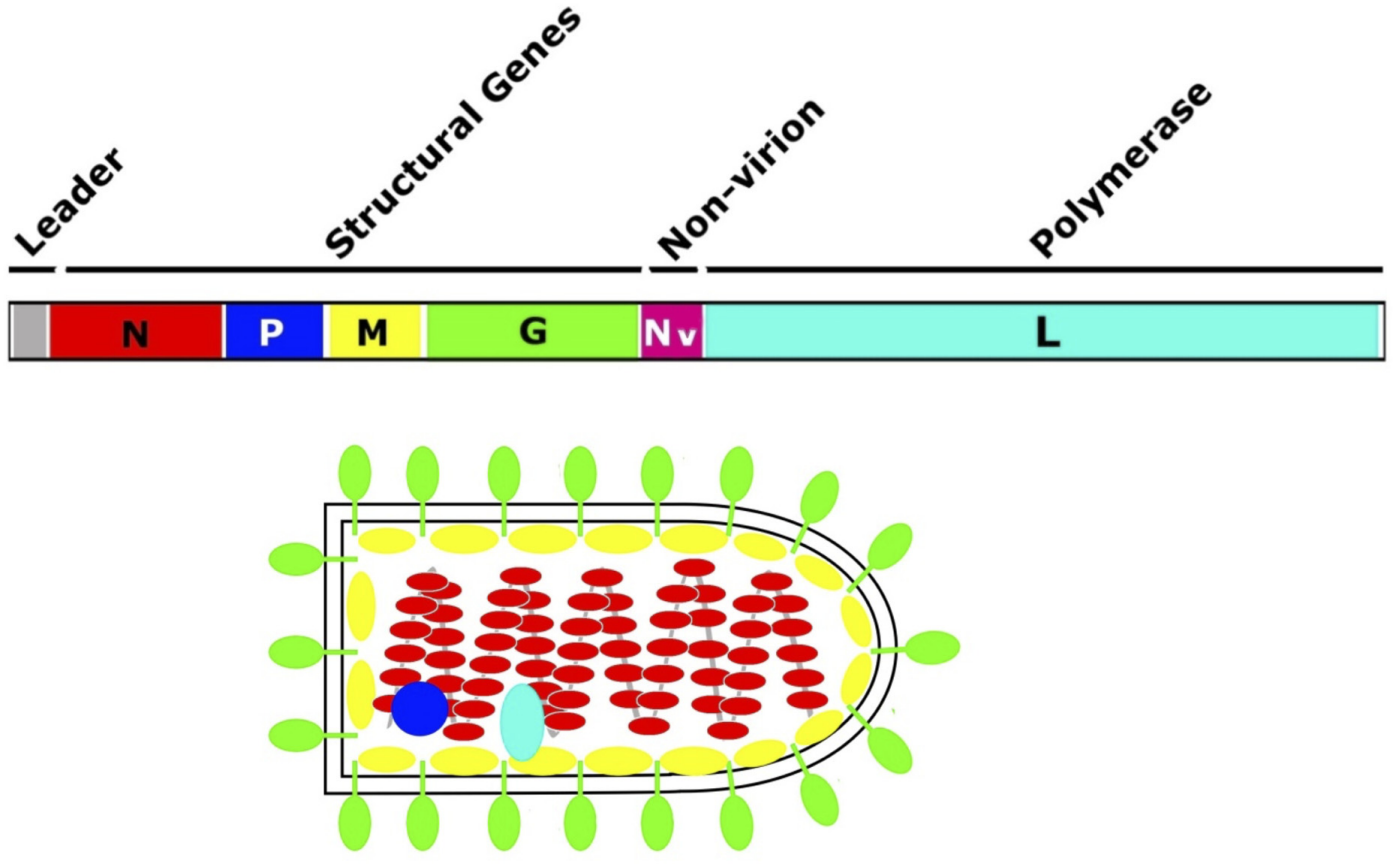
VHSv genome. Diagram of the VHSv genome, showing its six proteins and bullet-shaped virion structure (modified, with permission, from [21]).

The VHS rhabdovirus is bullet shaped, ~12,000 nucleotides (nt) in length, with six open reading frames of 3’*N-P-M-G-Nv-L*’5 (nucleoprotein, phosphoprotein, matrix protein, glycoprotein, nonvirion, and large protein; Fig 1). Fish deaths from VHSv have been reported to occur in water temperatures between 1–15°C [10] and to peak at 9–12°C [11]; the latter temperatures match those during the spring spawning aggregations of many Great Lakes' fishes. Encapsulated virus particles (virions) are transported via fish waste, reproductive fluids, or skin secretions, and can be further transported by boating, ballast water, and fishing tackle [12,13]. Virions have been reported to remain infectious to 13 days in water [14] and can travel up to two km [12], thus facilitating rapid and wide dispersal. Infected fishes often possess internal and external hemorrhages, pale gills, exophthalmia (bulging eyes), ascites (fluid in the peritoneal cavity), and/or erratic swimming [15,16].

VHSv belongs to the genus *Novirhabdovirus*, whose members exclusively infect fishes and possess the novel *Nv*-gene (421 nt). The International Committee on Taxonomy of Viruses [17] recognizes three other novirhabdoviruses: Hirrame rhabdovirus (HIRRv), Infectious Hematopoietic Necrosis virus (IHNv), and Snakehead rhabdovirus (SHRv). Other proposed novirhabdoviruses have included the Eel viruses B12 (EEv-B12) and C26 (EEv-C26), and the Rio Grande cichlid virus (RGRCv) [18]. The *Nv*-gene is believed to enhance viral replication and augment persistence of the virus in the host, prolonging shedding and thereby increasing spread of the virions to other hosts and geographic areas [19]. Although the *Nv*-gene has been discerned to evolve very rapidly in VHSv [2], little was known of its differentiation or mutational patterns prior to the present investigation.

The *G*- (1,608 nt) and *N*- (1,388 nt) genes of VHSv are less genetically variable than the *Nv*-gene [2], whereas the *P*-gene (760 nt) and the *M*-gene (741 nt) appear to be even more highly conserved. Glycoprotein (*G*) is believed to influence attachment to the host cell and induction of endocytosis [16]. Nucleoprotein (*N*) associates with negative- and positive-sense RNAs to modulate the balance between genome transcription and replication [20]. Phosphoprotein facilitates viral replication and inhibits activation of interferon response in the host cells. Matrix protein (*M*) inhibits activity of the interferon promoter [21] and assists in viral budding [22]. The RNA polymerase activity of the large protein encoded by the *L*-gene (6,087 nt) guides viral transcription and replication [16], and potentially plays a role in growth of the virus at different temperatures [23]. Here we compare relative substitution patterns and rates for the VHSv-IVb *G*-, *Nv*-, *P*-, and *M*- genes, in relation to their temporal and spatial evolutionary divergences across the Great Lakes. Phylogenetic trees of VHSv variants across its global distribution by Pierce and Stepien [2] showed that diversification tended to follow a “star-like” or “cloud-like” pattern. This evolutionary pattern may be suggestive of VHSv’s ability to infect multiple host species and enter new geographic areas.

**Fig 2:**
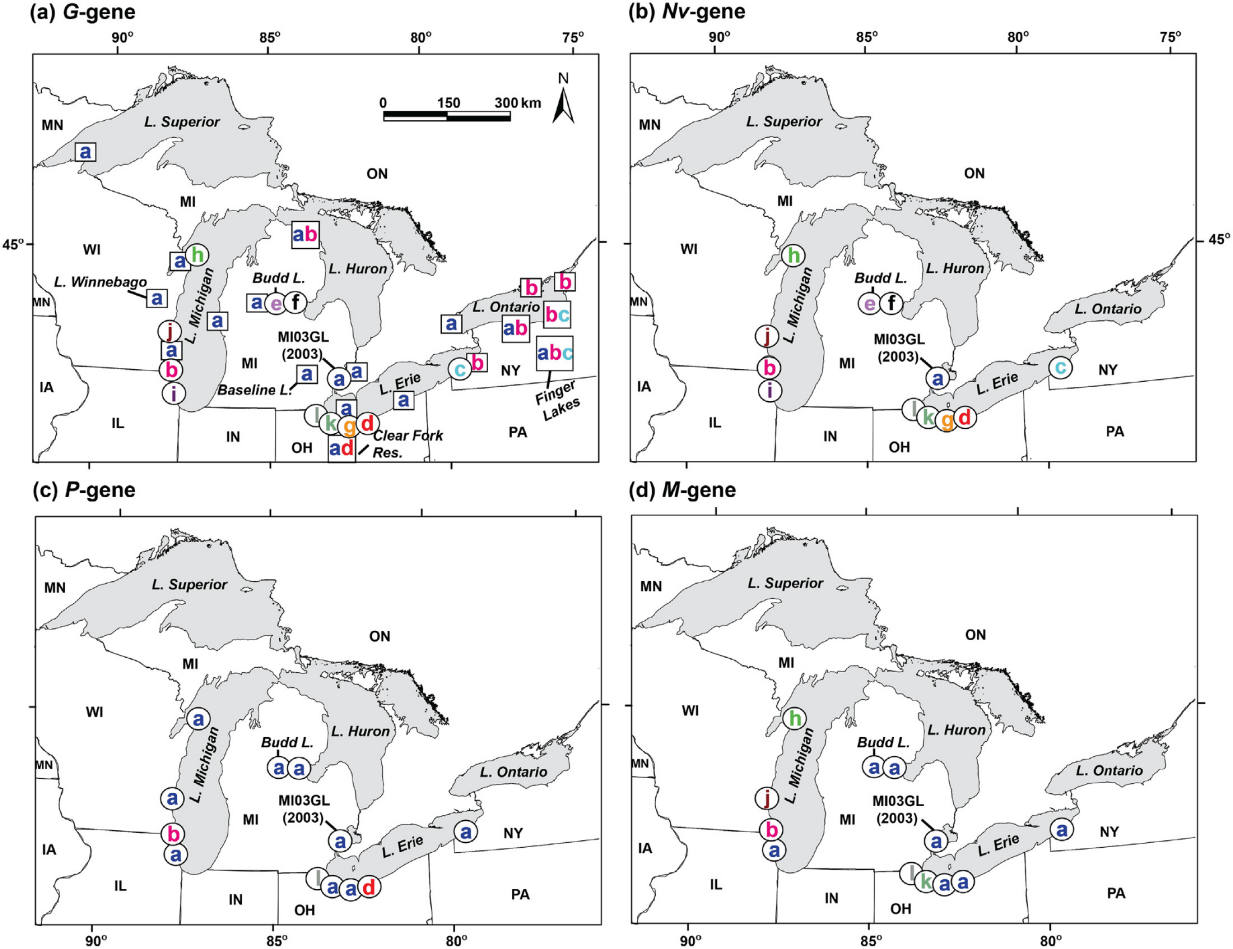
VHSv-IVb distribution. Maps showing the distribution of VHSv-IVb isolates and unique haplotypes (lettered according to Table 1) across the North American Laurentian Great Lakes for the (a) *G*- (b) *Nv*-, (c) *P*-, and (d) *M*- genes. Circles = isolates sequenced by us in the present study. Squares = individuals sequenced by other researchers and not available to this study. Maps were created with ArcGIS(R) software by Esri (Redlands, CA) and detail was added with Adobe Illustrator CC (Adobe Systems).

The first known occurrence of VHSv-IVb was traced back to a muskellunge (*Esox masquinongy*) captured from Lake St. Clair MI of the Great Lakes in 2003 ([5]; location marked on Fig 2). In 2005, large concurrent outbreaks killed muskellunge in Lake St. Clair MI [24], and freshwater drum (*Aplodinotus grunniens*) and round goby (*Neogobius melanostomus*) from the Bay of Quinte in northern Lake Ontario, Ontario, Canada [25]. The round goby is an invasive species that first appeared in the Great Lakes in 1990, has established large populations, and possesses substantial genetic diversity [26]; its population growth may have helped to spread VHSv-IVb—as the goby is highly susceptible to the virus [27]. The largest VHSv-IVb outbreaks to date occurred in 2006, spanning the lower Great Lakes (Lake St. Clair, the Detroit River, Lake Erie, the Niagara River, Lake Ontario, and the St. Lawrence River) and killing many fish species, including: muskellunge, freshwater drum, round goby, walleye (*Sander vitreus*), yellow perch (*Perca flavescens*), smallmouth bass (*Micropterus dolomieu*), largemouth bass (*Micropterus salmoides*), rock bass (*Ambloplites rupestris*), northern pike (*Esox lucius*), bluegill (*Lepomis macrochirus*), pumpkinseed (*Lepomis gibbosus*), black crappie (*Pomoxis nigromaculatus*), gizzard shad (*Dorosoma cepedianum*), common carp (*Cyprinus carpio*), silver redhorse (*Moxostoma anisurum*), shorthead redhorse (*M*. *macrolepidotum*), and lake whitefish (*Coregonus clupeaformis*) [24,28].

Following the initial large outbreaks of VHSv-IVb in 2005 and 2006, subsequent outbreaks occurred during 2007 [28] in several scattered inland lakes around Lake Michigan (including Budd Lake), as well as in the New York Finger Lakes, south of Lake Ontario (Fig 2a). In 2008, round goby and yellow perch died from VHSv-IVb in western Lake Michigan at Milwaukee Harbor WI [29]. Lake St. Clair MI was the site of another VHSv-IVb outbreak in 2009, which killed smallmouth bass [30]. Lake Superior has been free of outbreaks, however, in 2009 VHSv-IVb was detected in a lake herring (*Coregonus artedi*) from the Apostle Islands WI (Fig 2a) in Lake Superior [6]. No VHSv die-offs were reported in 2010, yet sampling described by Cornwell et al. [31] identified VHSv-IVb in four of the Great Lakes (all but Lake Superior), with 13% of 5,090 fishes sampled in 2010 testing positive. In 2011, IVb outbreaks occurred again in Milwaukee Harbor WI (yellow perch and yearling gizzard shad) and Budd Lake MI (smallmouth bass and sunfish) [32]. In 2012, a freshwater drum and a largemouth bass from central Lake Erie (at Sandusky OH) tested positive for VHSv-IVb in an assay performed by our laboratory [33,34]; its sequence results are reported here. No outbreaks were reported from 2012 to 2015.

The aim of the present study is to elucidate the evolutionary and biogeographic diversification patterns of VHSv-IVb in the Great Lakes. We analyze comparative evolutionary rates of four genes–*G*, *Nv*, *P*, and *M–*asking: (1) How much genetic change has occurred?, (2) How are the substitutions phylogenetically and geographically distributed?, and (3) Are these relationships consistent among the genes? Understanding how VHSv changes over time and across geographic locations may aid prediction of its future spread and virulence, and augment our understanding of rapid evolution by pathogens. As ancient and historic viral samples are becoming more accessible to science [35], in-depth evolutionary analyses are increasing our understanding and prediction of their spread, transmission patterns, and relative virulence, as well as host response. For example, serious human viral infections, such as Human Immunodeficiency Virus (HIV) [36] and Ebola Hemorrhagic Fever Virus [37], may show interesting parallels with animal pathogens, such as VHSv.

## Materials and Methods

### Samples, PCR amplification, and sequencing

We analyzed the mutational patterns of 12 VHSv-IVb isolates that have unique *G*-gene sequences across the Great Lakes region (labeled a–l in Table 1 and Fig 2), comparing patterns among four genes: *G*, *Nv*, *P*, and *M*. Viral RNA samples were obtained domestically and internationally by our lab under USDA veterinary permits 109231 and 119799, respectively. Ten isolates (isolates containing haplotypes labeled a–j) were provided to us as purified RNA (by G. Kurath, USGS, Seattle, WA), which were identified as unique for the *G*-gene by Thompson et al. [6], which had been determined from 108 individual fish and invertebrate samples representing a variety of locations (Fig 2, including: Lake Superior (one individual), Lake Michigan (7), Lake Huron (5), Lake St. Clair (14), Lake Erie (25), Lake Ontario (12), the St. Lawrence Seaway (24), and inland water bodies (20 individuals, from the states of WI, MI, OH, and NY, and the province of Ontario). We sequenced two additional viral isolates from a largemouth bass and a freshwater drum, which we identified as having new unique *G*-gene sequences (labeled k–l, respectively), after they tested positive for VHSv in our diagnostic assays [33,34]. Those two fishes, which were swimming erratically but lacked clinical signs of VHSv, were collected in April and May 2012 by the Ohio Division of Wildlife's (ODW) Fish Research Unit off their boat dock in central Lake Erie at Sandusky OH.

**Table 1:** *P*- and *M*-gene haplotypes and homologues for VHSv-IVb isolates having unique haplotypes for the *G*- and *Nv*- genes. Name of isolate, collection year(s), watershed(s), and homologous sequences are given (homologues are in parentheses). vcG designations denote central *G*-gene haplotypes identified by Thompson et al. [6].

Haplotype (letter) and isolate	Collection year of isolate (other year(s))	Watershed of isolate (others)	*P*-gene haplotype (homologue(s))	*M*-gene haplotype (homologue(s))
a. MI03GL	2003 (2006–9, 11)	L. St. Clair (all five Great Lakes)	a (c, e, f, g, h, i, j, l)	a (c, d, e, f, g, i)
b. TAVgr08-02 (vcG002)	2008 (2005–9)	L. Michigan (Lakes Huron, Erie, Ontario)	b	b
c. TAVgr07-12 (vcG003)	2007	L. Erie (L. Ontario)	(a)	(a)
d. TAvgr06-16 (vcG004)	2006 (2008)	L. Erie	d	(a)
e. TAVgr07-01 (vcG005)	2007	L. Huron	(a)	(a)
f. TAVgr07-04 (vcG006)	2007	L. Huron	(a)	(a)
g. TAVgr07-20 (vcG007)	2007	L. Erie	(a)	(a)
h. TAVgr07-24 (vcG008)	2007	L. Michigan	(a)	h
i. TAvgr08-03 (vcG009)	2008	L. Michigan	(a)	(a)
j. TAVgr09-17 (vcG010)	2008	L. Michigan	(a)	j
k. LMB2012*	2012	L. Erie	k	k
l. Drum2012*	2012	L. Erie	(a)	l
Total *N* haplotypes			4	6

* = collected for this study

The two fish specimens were euthanized with an overdose of 25 mg/ml tricaine methanesulfonate (MS-222; Argent Chemical Laboratories, Redmond, WA) and were sacrificed by the ODW, following University of Toledo Institutional Animal Care and Use Committee (IACUC) approved protocol #106419. The surgical site (anus to operculum) was disinfected with 100% ethanol and betadine using sterile equipment. The spleen was removed, placed in a 1.5 mL tube of RNAlater (Qiagen, Valencia, CA), and stored at -80°C under sterile conditions. Specimens were disposed of following our approved University of Toledo biohazard protocol.

Tissues were ground separately under liquid nitrogen with a sterile mortar and pestle. Their RNA was extracted following the TriREAGENT (Molecular Research Center Inc., Cincinnati, OH) manufacturer’s protocol, re-suspended in 30 μl RNase-free water, quantified with a NanoDrop 2000 Spectrophotometer (Thermo Fisher Scientific, Waltham, MA), and adjusted to 1 μg RNA/μl. RNA samples then were reverse transcribed to cDNA using 1 μg RNA, 5X First Strand buffer, 10 mM dNTPs, 0.05 mM random hexamers, 25 U/μl RNasin, and 200 U/μl M-MLV in 90 μl reactions (rxns). DNA-free DNase Treatment and Removal Reagents (Ambion Life Technologies, Grand Island, NY) were used to eliminate any contaminating gDNA. Reverse transcription was carried out at 94°C for 5 min, 37°C for 1 h, and 94°C for 5 min. The cDNA was stored at -20°C.

Each gene region was amplified using 2 μl cDNA (30–60 ng DNA template) in separate polymerase chain reactions (PCRs) containing 50 mM KCl, 1.5 mM MgCl_2_, 10 mM Tris-HCl, 50 μM of each dNTP, 0.5 μM each of the forward and reverse primers (Table A in S1 File), and 1 unit *Taq* polymerase (Promega, Madison, WI) in 25 μl reactions (rxns). PCRs included initial denaturation of 2 min at 95°C, followed by 30 cycles of 30 sec at 95°C, 30 sec at primer- and strain-specific annealing temperatures (Table A in S1 File), and 1.5 min at 72°C, with a final 2 min extension at 72°C.

PCR amplification products were visualized on 1% agarose mini-gels stained with ethidium bromide and then purified—either on gels using the QIAGEN Purification Kit or via PCR with a QIAGEN PCR Purification Kit (the latter was used in cases of less-efficient amplification). DNA sequencing was outsourced to Eurofins MWG Operon [http://www.operon.com/default.aspx]. Sequences were aligned and checked by us using BIOEDIT v7.05 [38], and then deposited in GenBank [http://www.ncbi.nlm.nih.gov/genbank/] as Accession numbers KF928905-29 and KR869651(Table B in S1 File), in fulfillment of data archiving guidelines [39]. Our final aligned sequences totaled 669 base pairs (bp) for the *G*-gene, corresponding to nt 3409–4077 of the full-length genomic sequence of Great Lakes index strain MI03GL [40] (GenBank accession no. GQ385941), 366 for the *Nv*-gene (nt 4580–4945), 541 for the *P*-gene (nt 1550–2090), and 564 for the *M*-gene (nt 2326–2889).

We additionally evaluated all available VHSv-IVb sequences for the *Nv-*, *P-*, and *M-* genes from GenBank and the literature, along with a representative subset of the 213 substrain IVa and five substrain IVc sequences available for the *G*-gene. Homologous and unique sequences were determined and are geographically referenced in Table B in S1 File. All sequences and their corresponding amino acids were compared to the whole VHSv-IVb genome sequence of the MI03GL muskellunge reference (labeled “a” in Table C in S1 File), which is the first known Great Lakes case. Representative sequences from VHSv European strains I (GenBank Accession #Z93412), II (#HQ112247, DQ159193, KF928925, KR869651 (the latter two were sequenced by us) and III (#EU481506) were employed as outgroups for rooting the phylogenetic trees (Table B in S1 File).

### Genetic diversity, sequence substitutions, and rates

We employed the definition of a haplotype as, “a unique gene sequence that differs by one or more nucleotide substitutions”–here in reference to the original MI03GL VHSv-IVb isolate, which we designate as haplotype “a”. Haplotypic diversity (*H*_D_—which measures the degree of variability of haplotypes in a population sample, and is an analog for heterozygosity [41,42]), numbers of nucleotide substitutions (transitions and transversions), and uncorrected pairwise (p-) genetic distances were calculated using ARLEQUIN v3.5.1.3 [43] and MEGA v5.0 9 [44]. Numbers of synonymous and non-synonymous changes were resolved with DNASP v5.10.01 [45]. Substitution types were plotted against p-distances in EXCEL (Microsoft Corp.); their correspondence to linear models was evaluated with *F*-tests [46] and their inter-relationships evaluated with ANCOVA in R v2.15.2 [47]. Student’s *T*-tests compared the relative numbers of substitution types among gene regions.

Tajima’s [48] *D* tests in ARLEQUIN and posterior probability analyses in OMEGAMAP [49] were used to evaluate for the possible influence of selection. OMEGAMAP also tested for possible recombination (*ρ*). Codon usage comparisons for OMEGMAP analyses were constructed using the coding sequence of the VHSv-IVb reference strain MI03GL in MEGA. The constant omega and constant rho models were used to evaluate possible selective pressure and/or recombination for each individual gene and the combined gene data sets, using the recommended improper inverse priors [49]. Two independent Markov chain Monte Carlo (MCMC) chains were run for 10 million iterations with sampling every 100 iterations, analyzed for stationarity, and merged after a 10% burn-in. The SUMMARIZE function in OMEGAMAP was used to estimate the posterior probability criterion for positive selection.

The numbers of nucleotide substitutions per site per year (*k* = substitutions site^–1^ yr^–1^) were determined from the p-distances, and overall divergence times were estimated using BEAST v1.71 [50] for the combined data set. All divergences were reported with the highest posterior density (HPD) determined by the program, which outputs a lower and higher year credible interval [50]. Amino acid substitutions were evaluated for each haplotype, with the reference haplotype a (MI03GL) used as a benchmark.

### Phylogenetic relationships and divergence patterns

Patterns of genetic relationships, spatial dispersion, and the number of evolutionary steps were evaluated using statistical parsimony haplotype networks in TCS v1.21 [51], with 500 steps set as the maximum. We tested the hypothesis of genetic differentiation (*θ*_ST_/1-*θ*_ST_) by geographic distance (measured as the natural logarithm of nearest waterway connections (km)) using Mantel tests in GENEPOP v4.2 [52]; regression significance was evaluated with 10,000 permutations. The nearest roadway distance was used for inland-locked Budd Lake (Clare County, MI); the virus may have been transported there overland via trailered boats, bait buckets, and/or fishing gear.

Phylogenetic relationships of VHSv-IVb variants were evaluated per gene with maximum likelihood (ML) in PHYML v3.0 [53] and Bayesian analyses in MRBAYES v3.2.1 [54]. Nucleotide substitution models were determined using the corrected Akaike Information criterion (AICc) in JMODELTEST v2 [55]. We thus employed the TPM1UF model [56] plus invariant sites (I = 0.6300) for the *G*-gene, K80 [56] for the *Nv*-gene, TVM [56] plus invariant sites (I = 0.4350) for the *P*-gene, and TIM1 [56] with a gamma (α) distribution (α = 0.3020) for the *M-*gene. ML nodal support was calculated from 2,000 nonparametric bootstrap pseudoreplications [57]. Bayesian analyses employed a Metropolis-coupled MCMC approach run for 5,000,000 generations, with sampling every 100 generations. Burn-in was determined by plotting log likelihood values to identify stationarity, discarding the first 25% (1,250,000) generations.

Combinability of the four gene regions into a single 2,140 nt data set for a “total evidence” phylogenetic analysis was supported by an Incongruence Length Difference test (ILD; [58]) in PAUP* v4.0 [59]; these data included our 12 VHSv-IVb isolates (Table 1), three strain IVa (isolates Makah, JF00Ehi1, and KJ2008; Table B in S1 File), and one IVc isolate (CA-NB00-02; Table B in S1 File), along with strains I (Hededam isolate, GenBank Accession #Z93412), II (isolate FI-ka663-06, and KR869651, sequence by us, for the *M*-gene), and III (isolate FA281107, EU481506 –the latter sequenced by us), which were used to root the tree. ILD employed 1,000 replicates in a parsimony framework, with critical α = 0.01; results supported combinability in a total evidence analysis (*p*>0.05), for which JMODELTEST selected the GTR model [56] with a gamma distribution (α = 2.6290) plus invariable sites (I = 0.4760). VHSv-IVb synapomorphies and autapomorphies were calculated using the scrolling bar in MEGA [44] and mapped onto the combined gene tree. Nucleotide differences are detailed in Tables C–D in S1 File.

Comparative divergence times for the overall data set were estimated from BEAST using the gamma distribution and invariant sites as calculated by JMODELTEST. BEAST model parameters followed a relaxed molecular clock with a lognormal distribution, and sampling every 100 of 10,000,000,000 generations. Outputs were assessed with TRACER v1.5 (in BEAST) to ensure that values reached stationarity. Collection dates were used as calibration points. Tree branches were determined in PHYML, with their Bayesian support values calculated from MRBAYES.

## Results

### Genetic diversity and substitutions

**Fig 3:**
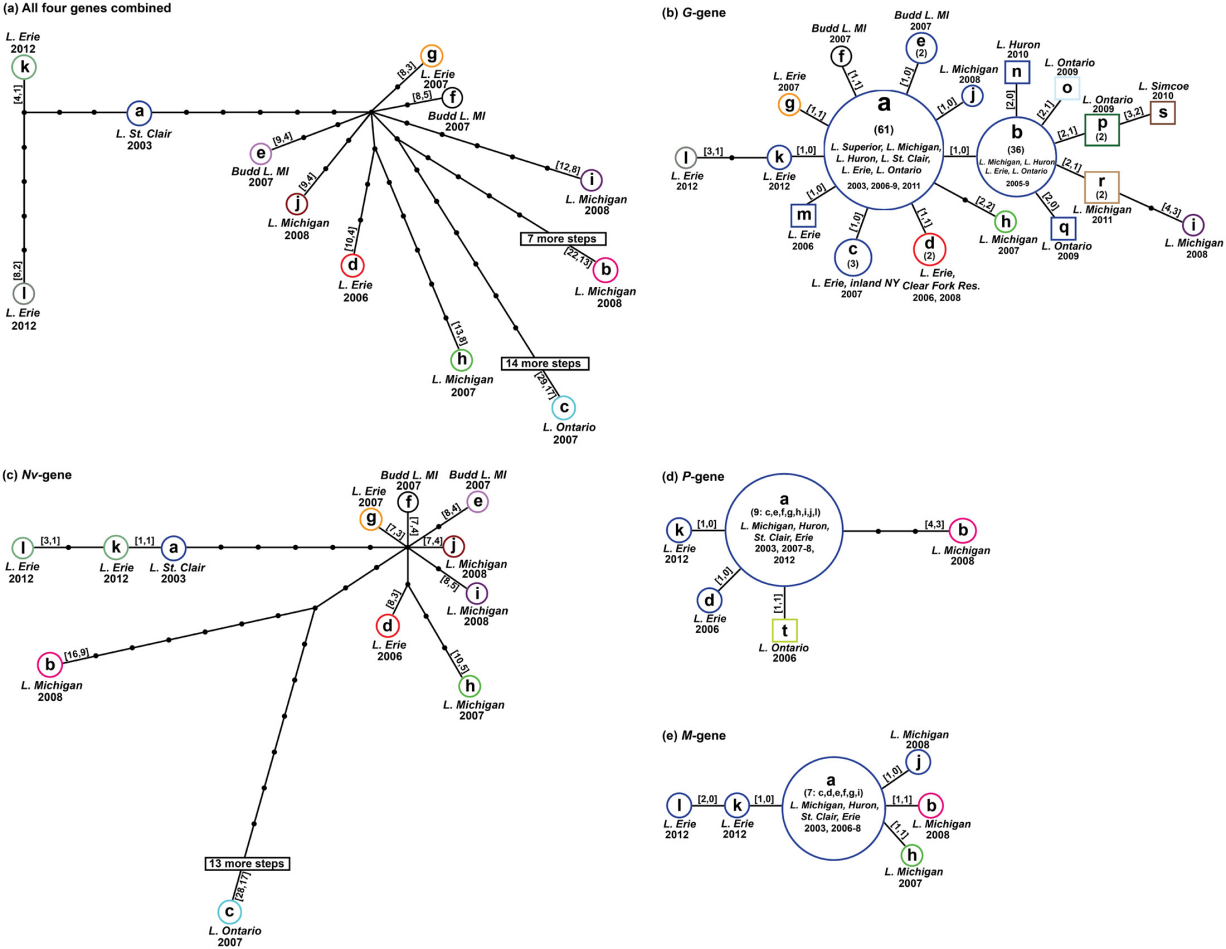
VHSv-IVb haplotype networks. Haplotype networks of VHSv-IVb variants from *TCS* 1.21 [39] for: (a) combined sequences of the four genes and for the (b) *G-*, (c) *Nv-*, (d) *P-*, and (e) *M-* genes. Circles are sized according to population frequency of the haplotype and lettered according to Table C in S1 File. Samples in squares were unavailable for sequencing by us (thus there are no data for the other genes). Colors surround haplotypes that encode a common amino acid. Lines denote a single substitution step between haplotypes; small, unlabeled black circles represent hypothesized haplotypes. Numbers in parentheses denote the total number of individuals sampled that share that haplotype (see Table B in S1 File). Numbers inside square brackets indicate the total number of nucleotide substitutions and number of changes in amino acids, respectively, compared to haplotype a. Collection location(s) and year(s) are listed beside each haplotype.

The 12 VHSv-IVb isolates are lettered a–l in Table 1. All had unique sequences for the combined gene and for the *G*-, and *Nv*- gene datasets (Tables 1–2 and Tables B–C in S1 File; Fig 3). Their respective haplotypes are designated as *C*-a through l (for the combined gene data), *G*-a through l, and *Nv*-a through l (Table 1). The number of *Nv*-gene haplotypes was constrained by the number of *G*-haplotypes, as we sequenced only those isolates that were variable in the *G*-gene. The 12 *G*-gene haplotypes differed by a total of 14 nucleotide substitutions, whereas there were 48 for the *Nv*-gene. Seven of those 12 *G*-gene haplotypes differed by single amino acid changes (encoding eight changes), and 11 *Nv* haplotypes encoded different amino acids, numbering 26 changes (Table 2; Fig 3 and Fig A in S1 File). The *P*-gene had just 33% of the total number of haplotypes relative to the *G*-gene (four total haplotypes: *P*-a, b, d, and k). Of these, only haplotype *P*-b encoded amino acid changes, diverging by three amino acids from haplotype a. The *M*-gene had six haplotypes (50% of the number relative to the *G*-gene: *M*-a, b, h, j, k, and l); all varied by single amino acid changes from haplotype *M*-a.

**Table 2:** Comparative genetic diversity values from 12 VHSv-IVb isolates for the *G-*, *Nv-*, *P-*, and *M*- genes.

	*G*	*Nv*	*P*	*M*
**Sequence Diversity**				
Sequence length (bp)	669	366	539	564
*N* of haplotypes (% of 12 isolates)	12(100%)	12(100%)	4(33%)	6(50%)
Haplotypic diversity (*H*_D_)	1.00±0.01	1.00±0.01	0.56±0.04	0.68±0.04
*N* of homologous sequences (%)	0(0%)	0(0%)	8(70%)	6(50%)
**Substitutions**				
*N* of substituted nucleotide sites (% of total bp)	14(2.1%)	48(13.1%)	6(1.1%)	5(0.9%)
Substitution rate: sub. site/yr (*k*)	2.0x10^-4^	2.8x10^-3^	1.2x10^-4^	1.2x10^-4^
*N* of 1st codon position substitutions (% of total)	4(29%)	14(29%)	2(33%)	0(0%)
*N* of 2nd codon position substitutions (% of total)	3(21%)	13(27%)	0(0%)	1(20%)
*N* of 3rd codon position substitutions (% of total)	7(50%)	21(44%)	4(67%)	4(80%)
*N* synonymous changes (% of substituted sites)	6(43%)	22(46%)	3(50%)	3(60%)
*N* non-synonymous (% of substituted sites)	8(57%)	26(54%)	3(50%)	2(40%)
**Divergence**				
Pairwise *N* of nucleotide substitutions between haplotypes: mean±SE (range)	2.8±0.4	10.7±2.3	2.5±0.7	1.9±0.3
	(1–7)	(1–31)	(1–5)	(1–3)
Pairwise (*p*) distances between haplotypes: mean±SE (range)	0.004±0.002	0.028±0.008	0.006±0.003	0.003±0.002
	(0.001–0.010)	(0.003–0.085)	(0.002–0.009)	(0.002–0.005)
Pairwise *N* of nucleotide substitutions between the 12 isolates: mean±SE (range)	2.8±0.4	10.7±2.3	1.0±0.4	0.9±0.4
	(1–7)	(1–31)	(0–5)	(0–3)
Pairwise (*p*) distances between the 12 isolates: mean±SE (range)	0.004±0.002	0.028±0.008	0.002±0.001	0.002±0.001
	(0.001–0.010)	(0.003–0.085)	(0.000–0.009)	(0.000–0.005)

All nucleotide positions were substituted just once, indicating lack of saturation in each of the four gene regions, i.e., there were no multiple “hits” (Tables C–D in S1 File). The *Nv*-gene had the most substitutions (48/366 positions, totaling 13.1%), followed by *G* (14/669, 2.1%), *P* (6/539; 1.1%), and then *M* (5/564, 0.9%) (Table 2). Most *Nv*-gene substitutions occurred at the third codon “wobble” position (21/48, 44%), greatly outnumbering those at the first (14/48, 29%) and second codon positions (13/48, 27%). The *G*-gene also had more nucleotide changes at the third codon position (7/14, 50%), than at the first (4/14, 29%) or the second (3/14, 21%). Most substitutions in the *P-* and *M-* genes occurred at the third codon position (4/6, 67%; 4/5, 80%, respectively). The *P*-gene had no changes at the second position and the *M-*gene had none at the first (Table 2 and Table C in S1 File).

**Fig 4:**
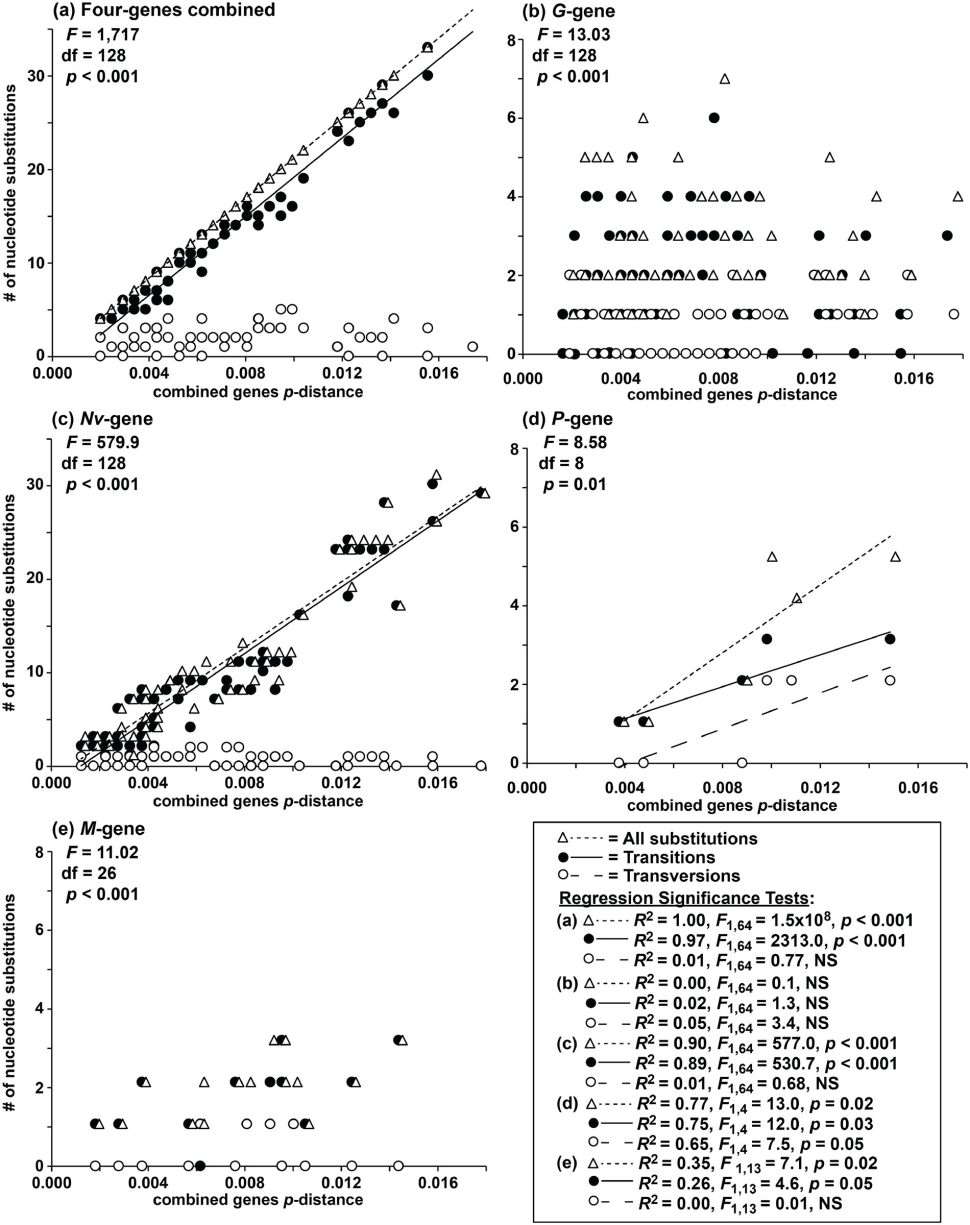
Tests for saturation. Numbers of nucleotide substitutions (triangle and small dashed line), transitions (black circle and straight line, and transversions (open circle and large dashed line), versus uncorrected pairwise (*p*-) distances of the combined sequence haplotypes for: (a) the combined sequences for the four genes and for the (b) *G*-, (c) *Nv*-, (d) *P*-, and (e) *M*- genes. Results from ANCOVA for the relationships of transitional and transversional substitutions are given under the gene heading. Regression lines are omitted when *R* ^2^<0.50.

Nucleotide substitutions increased linearly with p-distances in the combined gene data set (*R* ^2^ = 0.97–1.00, *F*_1,64_ = 2,313–1.5x10^8^, *p<*0.001; Fig 4a) and for the *Nv-* (*R* ^2^ = 0.89–0.90, *F*_1,64_ = 530.7–577.0, *p<*0.001; Fig 4c) and the *P-* genes (*R* ^2^ = 0.65–0.77, *F*_14_ = 7.5–13.0, *p* = 0.02–0.05; Fig 4d). Substitutions appeared level across the *G-* (Fig 4b) and *M*- genes (Fig 4e). Transitional substitutions significantly outnumbered transversions in the combined data set (*t* = 10.65, df = 130, *p*<0.001) and the *G-* (*t* = 5.96, df = 130, *p*<0.001), *Nv-* (*t* = 9.18, df = 130, *p<*0.001), and *M-* genes (*t* = 4.81, df = 28, *p<*0.001)–but not for the *P-*gene (Fig 4). Patterns of transitions and transversions did not appear directly related in any of the gene data sets (ANCOVA: *F*_8-128_ = 8.58–1,717, *p<*0.01–0.001).

**Fig 5:**
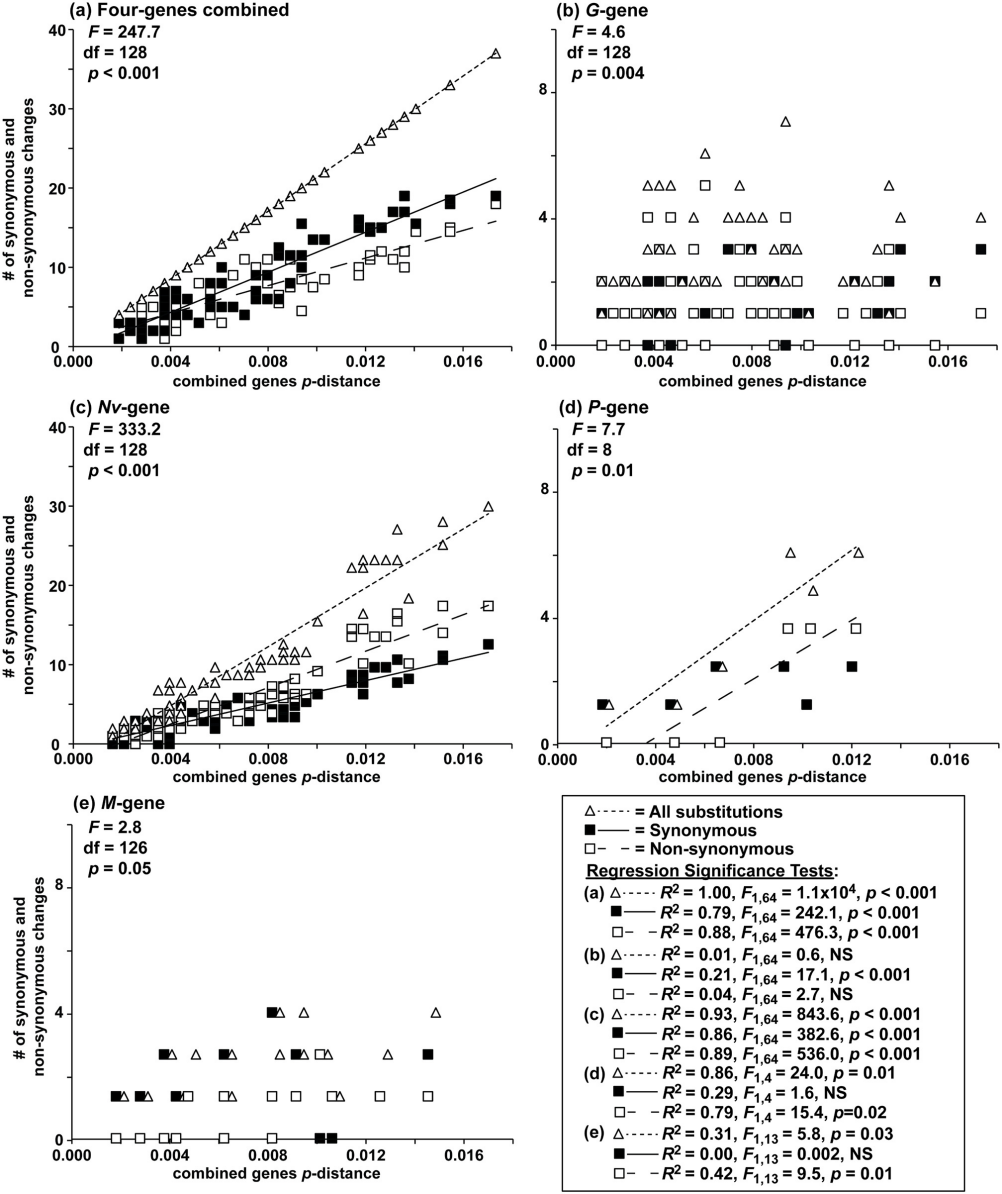
Coding vs. non-coding changes. Numbers of substitutions (triangle and small dashed line), synonymous (black square and straight line), and non-synonymous changes (open square and large dashed line), versus uncorrected pairwise (*p*-) distance of the combined sequence haplotypes for: (a) the combined sequences for the four genes and for the (b) *G*-, (c) *Nv*-, (d) *P*-, and (e) *M*- genes. ANCOVA results for synonymous and non-synonymous changes are located under each heading. Regression lines are omitted when *R* ^2^<0.50.

Numbers of synonymous and non-synonymous substitutions did not significantly differ (Fig 5), except in the *Nv*-gene (*t* = 2.02, df = 130, *p* = 0.045). Synonymous and non-synonymous changes displayed different trajectories, in relation to increasing p-distances (ANCOVA: *F*_8-128_ = 2.8–333.2, *p<*0.05–0.001). The *Nv*-gene had the most synonymous (22) and non-synonymous (26) changes, followed by the *G-* (6, 8), *P-* (3, 3) and *M-* (3, 2) genes (Table 2). Nucleotide substitutions increased linearly with p-distances in the combined data set (*R* ^2^ = 0.79–1.00, *F*_1,64_ = 242.1–1.10x10^4^, *p<*0.001; Fig 5a), and for the *Nv-* (*R* ^2^ = 0.86–0.93, *F*_1,64_ = 382.6–843.6, *p<*0.001; Fig 5c) and *P*- genes (*R* ^2^ = 0.79–0.86, *F*_1,4_ = 15.4–24.0, *p* = 0.01–0.02; Fig 5d). Substitutions in the *G-* (Fig 5b) and *M-* genes did not fit linear predictions (Fig 5d).

Tajima’s *D* values were negative and significant for all genes (combined data set *D* = -1.76, *p* = 0.03; *G*-gene *D* = -1.89, *p* = 0.004; *Nv*-gene *D* = -1.58, *p* = 0.05; *P*-gene *D* = -1.89, *p* = 0.01) except for the *M*-gene, indicating possible purifying selection. Analogous results were obtained from OMEGAMAP tests, showing the lack of positive selection (all posterior probabilities were less than 0.50: combined data set 3.5x10^-1^, *G*-gene 2.1x10^-1^, *Nv*-gene 3.9x10^-1^, *P*-gene 3.9x10^-4^, *M*-gene 4.4x10^-5^). Some possible recombination was inferred for the *Nv*-gene (*ρ*: 0.21, 0.06–0.73, 95% confidence interval) and the combined gene data set (*ρ*: 0.03, 0.01–0.07, 95%), but none for the *G*-, *M*-, or *P*- genes.

The overall evolutionary rate of VHSv-IVb was estimated as *k* = 4.8x10^-4^ for the combined data set. Individual gene rates within the Great Lakes were estimated as: *k* = 2.8x10^-4^ for the *G*-gene, *k* = 2.0x10^-3^ for the *Nv*-gene, *k* = 1.2x10^-4^ for the *P*-gene, and *k* = 1.2x10^-4^ for the *M*-gene.

### Genetic divergences and relationships among the haplotypes and isolates

Numbers of nucleotide substitutions varied among the four genes and were greatest for the *Nv*-gene, followed by the *G*, *P*, and *M*-genes (Table 2). Eleven of the 12 *Nv*-gene haplotypes varied in amino acids. The six nucleotide substitutions between haplotypes l and k (in the *G*-, *Nv*-, *P*-, and *M*-genes) only changed by one amino acid in the *G*-gene and the two haplotypes together shared an amino acid change (from the other 10 haplotypes) in the *Nv*-gene (Fig 3c and Fig Bc in S1 File). The 12 *G*-gene haplotypes were differentiated by 1–4 nt (Fig 3b), and four resulted in single amino acid changes, with haplotype h differing by two and haplotype i diverging by three amino acids, from haplotype a (Fig Ab in S1 File).

The *G*-gene haplotypes differed by an average of 2.8±0.04 pairwise nucleotide substitutions (mean uncorrected p-distance = 0.004±0.002; Table 2). The mean difference among *Nv*-gene haplotypes was ~3.6x greater, averaging 10.7±2.3 nt and diverging by 1–28 substitutions (mean p-distance = 0.028±0.008). The four *P-*gene haplotypes diverged by a mean of 2.5±0.7 nt, with 1–4 substitutions (mean p-distance = 0.006±0.003); haplotype b differed by three amino acids. However, since there were fewer *P*-gene haplotypes (four) for the 12 isolates, the mean difference among them was 1.0±0.4 nt (mean p-distance = 0.002±0.001). The *M*-gene had the fewest substitutions, differing by a mean of 1.9±0.3 nt among the six haplotypes (p-distance = 0.003±0.002), a mean of 0.9±0.4 nt among the 12 isolates (mean p-distance = 0.002±0.001), and with two separate single amino acid changes in haplotypes b and h.

Three isolates possessed unique nucleotide sequences for all four gene regions (Table C in S1 File): isolates a (MI03GL), b (vc002), and k (LMB2012). According to a geographic survey of *G*-gene haplotypes [6], haplotype *G*-a was the most widespread, occurring in all five Great Lakes (Fig 2a). *G*-b was the second most widespread, found in Lakes Michigan, Huron, Erie, and Ontario. We here identified two new unique isolates (k and l), which were discovered by us from single fish specimens in central Lake Erie. These both possessed unique *G*-, *Nv*-, and *M*- gene sequence haplotypes (Fig 3b and 3c). The two isolates differed by two nucleotides in the *G*- and *Nv*- genes, with haplotype l encoding a single amino acid change from haplotype a in the *G*-gene (Fig Ab in S1 File), and haplotypes k and l sharing an amino acid change from a in the *Nv-*gene (Fig Ac in S1 File). Isolate k additionally had a unique *P-*gene sequence and isolate l had a unique M-gene; neither resulted in an amino acid change (Fig Ad-e in S1 File).

In the combined gene haplotype network (Fig 3a), as well as the *Nv*-gene haplotype network (Fig 3c), haplotypes a, k, and l are located away from the others, with haplotype l being the most divergent. Six steps from haplotype a in those networks, nine haplotypes stem in a star-like pattern (b–j), with c being the most different. The *G*-gene haplotype network (Fig 3b) depicts abundant haplotype a as being central, from which nine other haplotypes differ by a single step. A second abundant haplotype, *G*-b, varies by just a single nucleotide from a, and also is located centrally; this does not encode an amino acid change from a. Haplotypes *G*-n, o, p, q, and r (o, p, and q from Lake Ontario in 2009–2010, n from Huron in 2010, and r from Michigan in 2011) form a cluster of distinct haplotypes one step away from b. Of these, o, p, and r encode amino acid differences (Fig Bb S1 File). The *P-* and *M-* gene haplotype networks likewise depict abundant haplotype a in the center (Fig 3d and 3e); the b-isolate differs in the amino acid sequence for each. All other *P-* and *M-* haplotypes stem from haplotype a by 1–2 steps, with the former diverging by an average of 2.5±0.7 nt and the latter averaging 1.9±0.3 (Table 2).

Similar relationships, as revealed among the haplotype networks (Fig 3; represented by colors and the second number on each branch), are apparent in the amino acid networks (Fig A in S1 File). The most noticeable alteration from the combined sequence haplotype network is the condensation of the split between *C-*k and *C*-l. In terms of amino acids, these 2012 isolates appear closer to haplotype *C-*a, with haplotype *C*-k having one amino acid change from *C*-a and two from *C*-l. *C*-b and *C*-c display the greatest number of amino acid changes, including (from *C*-a) for the *Nv-* (9 and 17), *P-* (3, 0), and *M*-genes (1, 0), respectively. Nucleotide differences and corresponding amino acid changes are indicated in Table C in S1 File.

**Fig 6:**
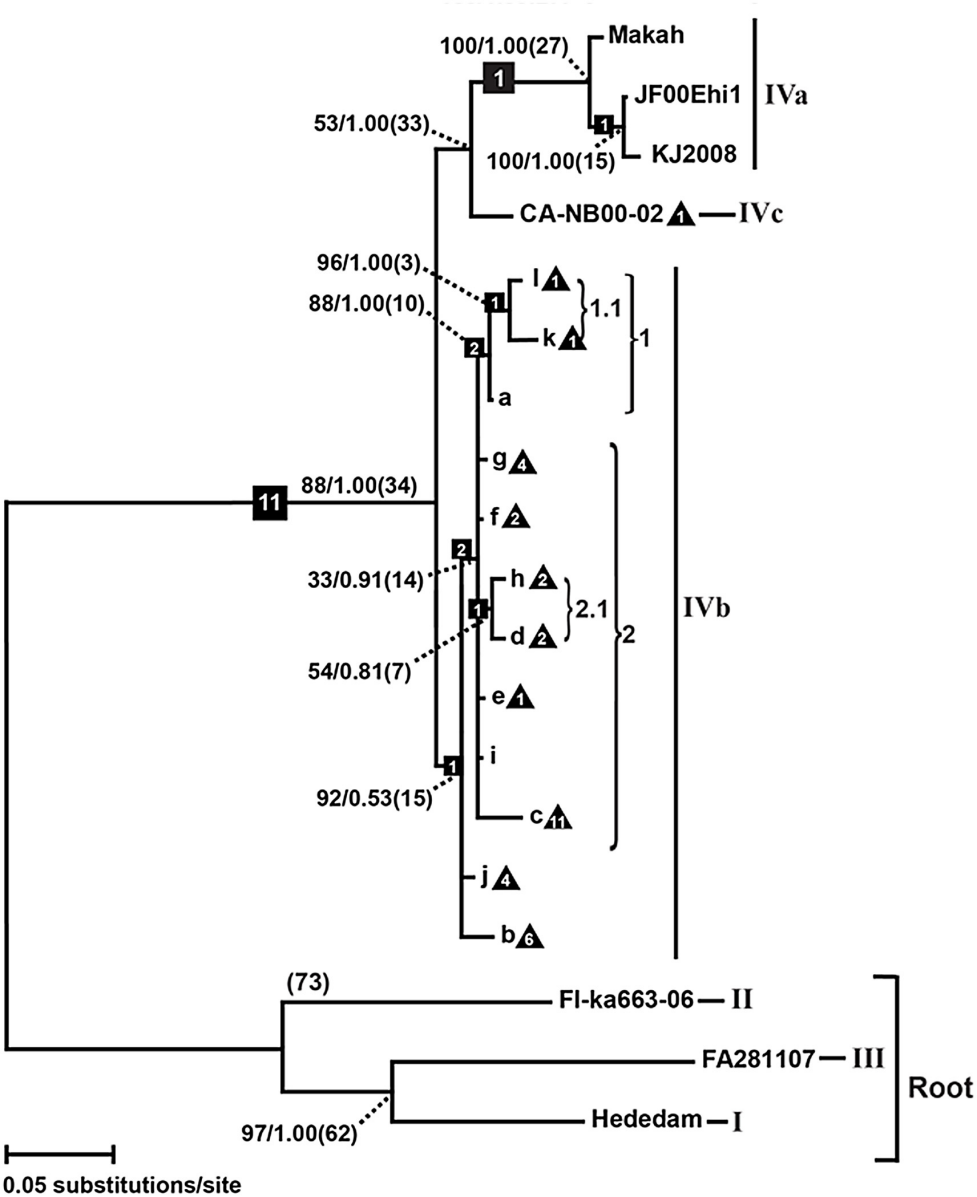
VHSv phylogenies. Phylogenetic tree of VHSv haplotypes based combined sequences for the *G-*, *Nv-*, *P-*, and *M-* genes (see Fig B in S1 File for individual gene phylogenies), from maximum likelihood and Bayesian analyses. Values above nodes = 2000 bootstrap pseudoreplicates/Bayesian posterior probabilities. Squares = number of synapomorphies; triangles = number of autapomorphies (see Table D in S1 File); parentheses = clades discussed in the manuscript. Estimated divergence times (years) are in parentheses. Trees are rooted to VHSv strain I, II, and III sequences.

Relationships among the combined haplotypes do not correspond to a genetic isolation by geographic distance pattern, according to results of the Mantel tests. The combined gene phylogeny shows two primary clades of VHSv-IVb (marked 1 and 2 on Fig 6), which are estimated to have diverged ~15 years ago (ya; 10–47 HPD). Clade 1 differentiated ~10 ya (9–16 HPD), with haplotype a as the sister taxon to a clade—labeled 1.1 –comprising haplotypes k and l. Haplotypes *C*-k and l differ from one other by six nt, diverging ~3 ya (0.002–9 HPD). Clade 2 was calculated to have originated ~14 ya (8–46 HPD) and contains the most haplotypes. Within it, haplotypes *C-*h (from Lake Superior) and d (from eastern Lakes Erie) comprise a sister group (2.1); these diverged from each other ~7 ya (6–16 HPD), are separated by 9 nt, and are geographically distant. Haplotype *C-*c is the most divergent in Clade 2, differing by 29 nt. All the other combined gene haplotypes vary by 8–22 nt from haplotype a.

Relationships in the *G*-gene tree (Fig Ba in S1 File) show a basal unresolved trichotomy for IVb, comprising haplotypes *G-*b and i, and clade 1 (containing haplotypes *G-*a, c–h, and j–l). Within clade 1, haplotypes *G-*k and l are sister taxa (labeled as 1.1; these are the 2012 samples from central Lake Erie), which diverge by 2 nt and 1 amino acid. Of the 14 unique *G*-gene nucleotide substitutions from haplotype *G-*a, eight (57%) resulted in amino acid changes (represented by different colors in Fig 3a–3e). All *G*-gene substitutions between haplotypes a, b, c, e, j, k, m, n, and q were synonymous and thus are colored with a single color on the haplotype network. Seven of the 12 *G*-gene haplotypes had a single amino acid change from haplotype a, except for haplotype *G*-h, which had two (Fig Ba in S1 File). Haplotypes m–s on the *G*-gene network (denoted by squares) were not available for us to sequence the other genes.

The *Nv*-gene tree (Fig Bb in S1 File) has two primary clades (labeled 1 and 2). Within clade 1, haplotypes *Nv-*k and l are sister taxa (labeled 1.1), which differ by two nt and comprise the sister group to haplotype *Nv-*a. Clade 2 contains haplotypes *Nv-*c–j in two internal subclades (labeled 2.1 and 2.2). Subclade 2.1 shows haplotypes *Nv-*d and h as sister taxa, which differ by four nt and were isolated one year apart from Lakes Erie (2006) and Michigan (2007), respectively (Fig 2c). Haplotypes *Nv-*c and b of subclade 2.2 markedly diverge by 26 nt, yet were collected just one year apart in Lakes Erie (2007) and Michigan (2008). Haplotype *Nv-*c is the most differentiated of the entire IVb *Nv*-gene phylogeny. There were 48 unique nucleotide changes in the *Nv*-gene, 26 (54%) of which changed the amino acid, while the remaining 22 unique substitutions (46%) were synonymous to the amino acid sequence of haplotype *Nv-*a. The haplotypes containing the largest number of nucleotide changes from haplotype *Nv-*a were found in haplotypes *Nv-*b (16 changes) and *Nv-*c (28), with 9 and 17 changes in the respective amino acid sequences. *Nv*-gene haplotypes l and k shared an amino acid substitution from haplotype a. The remaining haplotypes each differed from haplotype *Nv-*a from three to five amino acid changes (Fig Ab–c in S1 File).

The *P*-gene tree (Fig Bc in S1 File) does not display any major groupings for VHSv-IVb, and has just five haplotypes. Its haplotype *P-*b is the most divergent, by four nucleotides. Two haplotypes differed in amino acids from haplotype *P-*a (b and t; see Fig Ad in S1 File). Nucleotide substitutions in haplotypes *P-*k and d were synonymous with *P-*a. Haplotype *P-*b appeared most divergent, with three of the four nucleotide substitutions resulting in an amino acid change (Fig Ac–d in S1 File).

The *M*-gene tree (Fig Bd in S1 File) shows an unresolved relationship among VHSv-IVb haplotypes, with haplotypes *M-*k and l located together on an internal branch; these differ by a single nucleotide. Three other haplotypes differ by a single nucleotide from haplotype *M-*a; these were isolated from Lake Michigan fishes collected in 2007–2008. Of the five nucleotide haplotypes, two had non-synonymous substitutions that resulted in a different amino acid from *M-*a: haplotypes *M-*b and-h. These each contained a single unique amino acid substitution (Fig Ad–e in S1 File).

## Discussion

### Comparative substitution rates and diversity in the four genes (question 1)

VHSv-IVb appears to have differentiated extensively following its first appearance in the Great Lakes, with the *Nv*-gene evolving the fastest, at a rate ~7x faster (*k* = 2.0x10^-3^) than the *G*-gene (*k* = 2.8x10^-4^), and ~15x that of the *P-* (*k* = 1.2x10^-4^) and the *M-* (*k* = 1.2x10^-4^) genes. Our previous work estimated that the *N*-gene evolves at *k* = 4.3x10^-4^ across all four VHSv strains [2], ~3x slower than the *Nv*-gene.

The majority of the total VHSv-IVb *Nv*- and *G*-gene substitutions are non-synonymous (54% and 57%, respectively), resulting in functional protein changes that may be acted upon by selection. Among the 12 isolates tested, there are 26 amino acid changes among the 12 Nv-gene haplotypes, versus eight for *G*-, three for *P*-, and two for the *M*-gene. Tajima’s *D* tests [48] and OMEGAMAP posterior probabilities [49] suggest that the *Nv*, *G*, and *P* genes may be under purifying (negative) selection, i.e., the selective removal of deleterious alleles to maintain protein structure. Other studies have suggested that similar patterns of non-synonymous substitutions in RNA viruses are indicative of purifying selection [60]. Substitutions in the central *G*-gene sequences for the related *Novirhabdovirus* IHNv were attributed to purifying selection [61], however, subsequent analyses of its entire gene indicated possible positive selection instead [62]. Wu et al. [63] concluded that protein lengths, codon usage, gene expression, and protein interactions provide functional constraints to genetic changes—moderating evolutionary variation. Such differences among VHSv genes likely regulate their relative substitution rates and underscore the need for various pathogen challenge study models to examine the relationships among genetic composition, structure, and function.

Reverse genetic (knockout gene) experiments for the VHSv *Nv-*gene suggest that this gene has an anti-apoptotic role in early infection [64], which is thought to be an adaptive mechanism that lengthens the time the virus persists within the host. Increased duration of the infection can also increase the probability that new mutations will arise, due to the longer time spent in the host (and greater number of viral replications). For example, cell culture experiments by Biacchesi et al. [65] discerned that VHSv replication was reduced 10,000-fold when the *Nv*-gene was deleted. VHSv-IVb-challenged yellow perch experienced increased mortality compared to those infected by a genetically-altered strain in which *Nv* had been replaced with a green fluorescent protein, suggesting that *Nv* also functions in pathogenicity [19]. Similar findings showing *Nv*’s involvement in viral replication and pathogenicity have been reported for IHNv [65–67]. In contrast, replication and pathogenicity in mutant SHRv lacking an *Nv*-gene were analogous to those of the wild type [68,69]. This may indicate an evolutionary reversal in the Novirabdovirus lineage, in which the *Nv*-gene may have reverted to an ancestral "neutral" condition for SHRv. Among the Novirabdoviruses, SHRv appears more closely related to VHSv, whereas IHNv is more ancestral (see [2]). The high substitution rate of the *Nv*-gene in the VHSv-IVb substrain suggests it has high tolerance of functional substitutions. A viral population that possesses such wide variety of variants may experience enhanced survival in host populations, as well as greater ability to infect new ones. These hypotheses merit experimental testing.

The current study estimates the *G*-gene’s rate of substitution at ~2.0x10^-4^, which is similar to that calculated by Pierce and Stepien [2] across all VHSv *G*-gene strains. This rate is less than that reported for the distantly related rabies virus (~4.1x10^-4^) by Holmes et al. [70]. The present calculated substitution rate for the VHSv-IVb *G*-gene substrain IVb is slower than estimates for VHSv strains overall reported by Benmansour et al. [71] (1.2x10^-3^) and Einer-Jensen et al. [72] (1.74x10^-3^–7.06x10^-4^). These disparities may be due to differences in the regions that were sequenced, among the various strains/substrains and isolates that were selected, and/or the practice of partitioning sequences into freshwater versus marine viral subgroups in these studies [71,72].

The *G-*gene encodes glycoprotein, which forms spike-like projections on the viral particle surface (Fig 1) that attach to a host’s cell by binding to cellular receptors, including fibronectin [73] and perhaps other cell membrane structures such as heparin-sulfate, etc. Glycoprotein also has an antigenic role, eliciting pronounced host immune responses [16]. Changes in the *G*-gene may facilitate virus adherence and entry into the host's cells, in the face of the latter’s ability to recognize the virus and circumvent that entry. *G*-gene mutations thus may contribute to the virus’ evasion of the host’s innate and adaptive immune responses.

Transitional substitutions greatly outnumbered transversions in all gene regions analyzed except for the *P*-gene, indicating that their phylogenetic signals appear relatively free of saturation (see [74,75]). These ratios were similar across all four VHSv strains for the *G-* and *Nv*- genes [2], with proportional differences from the present study due to exclusive analysis of the IVb substrain.

The *P-*protein functions in VHSv viral replication [64] and may contribute to inhibition of host innate immune response [21]. Similarly, the *P*-protein of the distantly related rabies virus inhibits host innate immune signaling via interferon regulatory factors (IRF) and interferon signaling intermediates [76]. Our study shows that the *P-*gene has low sequence divergence, few amino acid changes from haplotype a, and evolves slower than the *Nv-* and *G-* genes. This lower rate of substitution further suggests that mutations in the *P-*gene may be deleterious to viral replication within the host.

The *M*-gene, like the *P*-gene, also appears to evolve slowly; M has six unique sequence haplotypes, with two having single amino acid changes. The *M*-protein interacts with the *G*-protein to assist in viral budding [22]. In Vesicular Stomatitis virus (VSv), another rhabdovirus, the *M*-protein appears to block gene expression [77] and/or inhibits export of mRNA from the nucleus, effectively blocking translation of host proteins—including those thought to be involved with the viral response pathway [78]. Mutations in the *M*-gene might lessen interference with the host gene expression machinery and thus increase competition for protein translation in the cytoplasm. This could reduce viral replication or enhance host innate responsiveness. Reverse genetic studies are needed to test these hypotheses.

### Geographic patterns and phylogenetic relationships from *G*-gene sequences (question 2)

Lake Erie houses the greatest VHSv-IVb *G*-gene richness (eight haplotypes, five of which appear unique; 63%), followed by Lakes Ontario (seven, three; 43%), Michigan (six, four; 67%), Huron (three, one; 33%), St. Clair (one, zero), and Superior (one, zero), based on our sampling and other efforts [79,80]. Lake Erie is the warmest and shallowest of the Great Lakes [81], and its warmest and shallowest western basin is located relatively close to the original VHSv-IVb isolate “haplotype a” (MI03GL) described from Lake St. Clair. VHSv-IVb differentiation may have been facilitated by Lake Erie's warm water temperatures and high fish abundances, estimated to contain ~50% of all Great Lakes fishes (Dr. Jeffrey Reutter, Ohio State Univ., pers. commun.). Kane-Sutton et al. [13] analyzed yellow perch from central Lake Erie in 2007 and 2008, discerning that VHSv-IVb infection was greatest in water temperatures of 12–18°C and at higher fish densities, such as during spawning aggregations. The 2006 VHSv-IVb outbreak occurred in June, at surface water temperatures of ~15–16°C, when many fishes—including yellow perch—were spawning.

Haplotypes a and b are the oldest known (first isolated in 2003 and 2005, respectively), most abundant (61, 36 sequenced occurrences), and widespread. Halotype a (MI03GL) has been found in all five Great Lakes, and haplotype b in all but Lake Superior (Fig 2a). Haplotype b first was identified during the 2005 Bay of Quinte, Lake Ontario outbreak. Haplotypes a and b do not appear closely related, according to their *Nv*- and *P*- gene sequences, and differ by a single step in the *G*- and *M*- gene haplotype networks. Moreover, b also is distinctively divergent in amino acids from a in its *Nv*-, *P-*, and *M*-genes, but is identical to a in the *G*-gene. The two comprise the center nodes in the *G*-gene haplotype network, with many other haplotypes diverging from them. Haplotype b may have differentiated from a, subsequent to the first discovery of VHSv-IVb in the Great Lakes. Alternatively, according to the *Nv*-gene networks, both haplotype a and haplotype b may have separate, earlier origins that predate their discovery in the Great Lakes. This latter hypothesis may be supported by their amino acid differentiation.

Eleven *G*-gene haplotypes (b–h, j–m) appear closely related to the original a haplotype (Fig 3b). Two descendants of haplotype a:–haplotype d in western Lake Erie OH and m in central Lake Erie—appear to date back to the 2006 outbreak. Five haplotypes first were found in the 2007 outbreak (e and f from the outbreak in Budd Lake MI, h in northwest Lake Michigan, g in western Lake Erie, and c in eastern Lake Erie NY and south of Lake Ontario in the inland NY Finger Lakes). Haplotype j was identified from the 2008 outbreak near Milwaukee WI in Lake Michigan. Seven haplotypes (i, n–s) appear to have descended from haplotype b, and were identified from samples collected in 2008 (i, in the Milwaukee WI Lake Michigan outbreak), 2009 (o, p, and q in eastern Lake Ontario), 2010 (s from Lake Simcoe, ON, and n in northwest Lake Huron), and 2011 (r, from Lake Michigan in 2011). Occurrence of new *G*-gene nucleotide variants stemming from haplotypes a and b suggests that the virus likely was mutating after the 2006 outbreak throughout the Great Lakes; this evolution may have been in response to the host populations developing resistance to VHSv following the large fish die-offs. In this study, we describe two new haplotypes, k and l, sampled from central Lake Erie, near Sandusky, OH in 2012. Haplotype l may have descended from k, as shown in the *G-*gene haplotype and amino acid networks (as well as the *Nv-* and *M*- haplotype networks; Fig 3 and Fig A in S1 File). Continued evolution of new *G*-gene variants may facilitate the virus’ ability to avoid recognition by new host individuals despite previous exposure of the host population to other viral mutants.

### Evolutionary patterns: consistency and differences among the genes (question 3)

Prior studies mostly have concentrated on the evolutionary patterns of VHSv’s *G-*gene [2,6,7], which has over 300 haplotypes across the four strains. The present study employs a more rigorous phylogenetic approach to examine multiple genes (*G*, *Nv*, *P*, and *M*), providing a more robust analysis of VHSv-IVb evolutionary patterns. All four genes in this study display star-like radiations that follow a quasispecies model, depicting a rapid evolutionary burst that may underlie VHSv-IVb’s spread into new geographic areas, habitats, and hosts [2]. In the *Nv*-gene network (Fig 3c), haplotypes a, k, and l appear distinct from the others (the latter two also differing by an amino acid substitution), for which the k and l isolates were identified by our team from central Lake Erie in 2012. Haplotypes k and l were not linked to an outbreak, but denote continued evolution of *Nv*-gene variants, which may allow these newer haplotypes to persist longer in fish hosts and invade new ones. Mutations in such highly conserved genes may increase the ability of VHSv-IVb to circumvent the innate immune response of fish populations. This merits further testing.

The *Nv* haplotype and amino acid networks show two other haplotypes, b and c, which also are very different from the others (Fig 3c and Fig Ac in S1 File). Haplotype b appears distinct for the *G-*, *Nv-*, *P-*, and *M*-genes in the nucleotide networks; it is located at the center of the *G*-gene network and may be one of the oldest and original VHSv-IVb variants. Its *Nv-*, *P-*, and *M-*genes moreover differ in amino acids. Haplotype c matches haplotype a in both the *P-* and *M-* genes, and occurs in eastern Lake Erie and the New York Finger Lakes south of Lake Ontario.

In both the *Nv* haplotype and amino acid networks, a distinct and closely related cluster of seven haplotypes (d–j), traces its differentiation to 2006–2008 in Lakes Erie and Michigan. This cluster contains two Lake Erie haplotypes (d and g), which respectively date to the 2006 outbreak and 2007. Members of the *Nv* cluster appear to have been very successful in the 2007 Budd Lake and the 2008 Milwaukee, Lake Michigan outbreaks. All of these *Nv* cluster members thus appear to be evolutionarily related—stemming from a common *Nv* substitution six nucleotide steps from isolate a—and rapidly mutating in a star-like cluster, with 1–4 additional substitutions and all differing in amino acids. In the *G*-gene haplotype network, six of these *Nv*-gene cluster members (all except for i) descend from the central *G*-a haplotype; all but *G*-e and *G*-j have amino acid differences. A single member of the cluster—d—also had a synonymous change in the *P*-gene (from western Lake Erie in 2006). Two others had *M*-gene substitutions—h and j (from Lake Michigan, in 2007 and 2008), with the former being non-synonymous, and the latter synonymous.

Of the 12 isolates tested, haplotypes a and b are located centrally in the *G*-gene haplotype network, with other haplotypes stemming from them. In the *Nv* and *P* haplotype networks, haplotypes a and b appear quite distant in nucleotide sequences, as well as amino acids, diverging by nine and three amino acids, respectively. These two haplotypes also possess different *M*-gene sequences and amino acids.

Haplotypes k and l, collected in 2012 from central Lake Erie fishes, are evolutionary divergent from the other haplotypes in three of the gene sequences (*G*, *Nv*, and *M*), and are closely related to each other. They diverge from each other by just a single *G*-gene amino acid. A cluster of seven *Nv*-gene haplotypes (d–j) identified in 2006–2008 from Lakes Erie and Michigan radiates outward from a central hypothesized *Nv-* haplotype that is separated by six steps from the original haplotype a sequence. In the *G*-gene network, six of these haplotypes descend directly from haplotype *G*-a d–h and j), whereas just d of this group also is differentiated for the *P*-gene and for the *M*-gene just h and-i are distinct. In summary, haplotype mutational patterns appear to coincide among genes in some cases, but seem to have changed independently for other VHSv-IVb variants.

It is interesting to note that haplotypes k and l, first analyzed in this study, were collected from the same location (nearshore in Sandusky OH, central Lake Erie), just one month apart in 2012, yet have unique sequences for the *G-*, as well as the *Nv-* and *M*- genes. Together they share a differentiated amino acid (for the *Nv*-gene), with l also differing in a *G*-gene amino acid. Both fishes had no outward lesions or internal hemorrhaging, and appeared free of clinical signs of infection, in contrast to those that characterized the 2006 outbreak in Lake Erie. Their sole symptoms were disorientation and erratic swimming. Other fishes in the area did not appear affected. Monitoring [82,83] and sequencing studies [6,30] similarly have reported that some Great Lakes fishes that tested positive for VHSv lacked clinical signs of the disease. For example, Frattini et al. [82] surveyed fishes for VHSv in fall—winter 2006, following the summer 2006 outbreak, throughout the state of New York (in eastern Lake Erie, Lake Ontario, and inland lakes). Of the 1,011 fishes sampled, 69 (6.8%) tested positive and all lacked visual VHSv symptoms. Tests of Lake Erie yellow perch in 2007–2008 showed that 60% of pooled samples tested VHSv positive, yet appeared symptom-free [13]. Thompson et al. [6] analyzed 108 fishes, along with leech (*Myzobdella lugubris* and amphipod *Diporeia* spp.) invertebrates sampled in 2003–2009 across the Great Lakes, finding that 28 (26%) of the individuals were positive, but asymptomatic. Cornwell et al. (2015) also discerned VHSv-IVb in samples collected from Lakes Michigan, Huron, Erie, and Ontario in 2010 [31]. These studies lend support to the hypothesis that VHSv-IVb is prevalent across the Great Lakes, but may have become less virulent over time.

Our findings suggest that IVb may have responded by mutating into a variety of variants to aid evasion of host immune responses. Similarly, some human viruses undergo rapid evolution; for example, the Ebola Hemorrhagic Fever virus has displayed a high degree of genetic variation, and was estimated to be evolving at a rate of *k* = 9.6x10^-4^, somewhat similar to our estimate for VHSv [84,85]. These hypotheses of rapid VHSv evolution merit further surveillance efforts and challenge studies (*in vitro* and *in vivo*) to interpret virulence.

### Conclusions

Understanding the evolutionary history of rapidly evolving viral sequences is dependent upon fine-scale study of specific genetic changes in individual genes at the population level [1], as we have begun to undertake in this analysis. This is the first study to report and analyze sequence variants for the *Nv-*, *P-*, and *M-* genes of VHSv-IVb. Our results show that the most rapid differentiation occurred in the *Nv-*gene, which is unique to the novirhabdoviruses. The *G*-gene evolved at ~1/7th the rate of *Nv*, with the *P-* and *M*- genes at ~1/15 the rate of *Nv*. Some viral variants displayed roughly similar divergence patterns among the different genes, whereas others appeared independent. Much genetic differentiation followed the widespread 2006 outbreak, reflecting possible response of the viral population to host population resistance. Two new, unique, and divergent viral variants occurred in fishes from central Lake Erie in 2012; these lacked classic disease symptoms, indicating ongoing mutational change. Rapid evolutionary diversification may allow VHSv to evade fish host recognition and immune response, facilitating endemic maintenance in populations—as well as new colonizations. These findings aid understanding of the co-evolutionary patterns of a virus and its hosts, and may assist prediction of future spread and success.

## Supporting Information

S1 File VHSv primer sequencing and sample information, and amino acid and phylogenetic findings. Primer sequences used for our PCR and sequencing reactions (Table A). VHSv samples used in our analyses (Table B). Sequence specifics for unique VHSv-IVb haplotypes (Table C). VHSv-IVb sequence characters, based on the *G-*, *Nv-*, *P*-, and *M*-gene sequences (Table D). Amino acid networks (Figure A). VHSv phylogenies (Figure B).
(DOCX)
