# Supplementary material for: Gene Diversification of an Emerging Pathogen: A Decade of Mutation in a Novel Fish Viral Hemorrhagic Septicemia (VHS) Substrain since Its First Appearance in the Laurentian Great Lakes
Source: PLoS One. 2015 Aug 27;10(8):e0135146. doi: 10.1371/journal.pone.0135146 (PMC4552161; doi:10.1371/journal.pone.0135146)
Supplement: S1 File — Primer sequences used for our PCR and sequencing reactions (Table A). VHSv samples used in our analyses (Table B). Sequence specifics for unique VHSv-IVb haplotypes (Table C). VHSv-IVb sequence characters, based on the G-, Nv-, P-, and M-gene sequences (Table D). Amino acid networks (Figure A). VHSv phylogenies (Figure B). (DOCX) [file pone.0135146.s001.docx]

**Supplementary File (Tables A**–**D, Figures Aa**–**e, Ba**–**d)**

**Table and Figure Legends**

**Supplementary Table A. Primers used for PCR and sequencing per gene, with name, reference, sequence, annealing temperature, and extension time.**

**Supplementary Table B. VHSv samples used in our analyses.** VHSv strain, host species, isolate, GenBank accession number, homologous sequences, reference, and locality per gene sequence (*G, Nv*, *P*, *M*) . *=sequenced by us in this study. (for coordinates) = unreported in reference.

**Supplementary Table C. Sequence substitutions and amino acids of unique VHSv-IVb haplotypes for the (a) *G-,* (b) *Nv-,* (c) *P-,* and (d) *M-* genes.** Top: Nucleotide position in reference to aligned GenBank sequence, followed by position in M103GL (haplotype a) complete genome sequence (in parentheses), and codon position place (1, 2, or 3). Left column: haplotypes (lettered a-1) and their isolates (along with sequence identifiers used for the *G*-gene by Thompson et al. [6], in parentheses). Column 2: GenBank accession number. Main body: Nucleotides (G, A, T, C) per position (top) in reference to the baseline M103GL sequence (haplotype a), followed by the amino acid (in parentheses) . = nucleotide and amino acids are identical to MI03GL. * = described in this study. ^+^ = indicates nucleotide change results in different amino acid than MI03GL (haplotype a).

**Supplementary Table D. VHSv sequence characters for the *G-, Nv-*, *P*-, and *M*-genes.** Nucleotide positions are numbered according to the complete IVb MI03GL isolate (=haplotype a). Colors denote characters that distinguish haplotypes and their clades. Green= apparent synapomorphies for strain IV, yellow = strain IVb, pink = other clades. Autapomorphies = aqua. Grey = ambiguous characters (apparent homoplasies and/or reversals). Red = Single or linked reversals.

**Supplementary Figure A.** **Amino acid networks.** Amino acid networks of VHSv-IVb variants for: (a) combined sequences of the four genes and for the (b) *G-*, (c) *Nv-*, (d) *P-*, and (e) *M-* genes. Circles are sized according to population frequency of the amino acid sequences and lettered according to Table C in S1 File. Samples in squares were unavailable for sequencing by us (thus there are no data for the other genes). Lines denote a single substitution step between haplotypes; small, unlabeled black circles represent hypothesized amino acid sequences. Numbers in parentheses denote the total number of individuals sampled that share the amino acid sequence (see Table C in S1 File). Collection location(s) and year(s) are listed beside each haplotype.

**Supplementary Figure B.** **VHSv phylogenies.** Phylogenetic trees of VHSv haplotypes based on the (a) *G-*, (b) *Nv-*, (c) *P-*, and (d) *M-* genes, from maximum likelihood and Bayesian analyses. Values above nodes = 2000 bootstrap pseudoreplicates/Bayesian posterior probabilities. Parentheses = clades discussed in the manuscript. Estimated divergence times (years) are in parentheses. Trees are rooted to VHSv strain I, II, and III sequences.

**Supplementary Table A**

| Gene | Primer Name | Reference | Sequence (5’-3’) | Annealing Temp. (^o^C) | Extension Time (min) |
| --- | --- | --- | --- | --- | --- |
| *G* | Gint1F | [6] | TCCCGTCAAGAGGCCAC | 53 | 2:30 |
|  | Gint4R | “ ” | TTCCAGGTGTTGTTTACCG | ” | ” |
| *Nv* | VHSvNv.se | [21] | ACGAATTCATGACGACCCAGTCGGCAC | 57 | 1:30 |
|  | VHSvNv.as | “ ” | ACGGTACCTGGGGGAGATTCGGAGCCA | ” | ” |
|  | MI03GLNv.for | This study | GCACCCCTGTGAGACAGAAA | 55 | ” |
|  | MI03GLNv.rev | “ ” | TGGGAGAAGGGGGAGGAG | ” | ” |
| *P* | VHSvPFor2 | This study | CGCTGAGAGCTCACAATGAC | ” | 2:00 |
|  | VHSvRev2 | “ ” | GCCTTGATTGCCTTTGAGAC | ” | ” |
| *M* | VHSvM.se | [21] | ACGAATTCATGGCTCTATTCAAAAG | ” | ” |
|  | VHSvM.as | “ ” | ACGGTACCCCGGGGTCGGACAGAG | ” | ” |

**Supplementary Table B**

| Gene | Strain | Host Species | Isolate | Accession No. | Homologous Sequences: Isolate (Accession No.) | Reference(s) | Locality | Lat. | Long. |
| --- | --- | --- | --- | --- | --- | --- | --- | --- | --- |
| ***G*** | **I** | *Oncorhynchus mykiss* | Hededam | Z93412 | __ | [86] | Spjarup Hededamme, DNK | 56.648 | 9.271 |
|  | **II** | *Clupea harengus* | ka663-06 | HQ112247 | ka664_06 (HQ112231)  ka662_06 (HQ112230) | [87]  “ “  “ “ | Archipelago S., FIN  “ “  “ “ | 60.290  “ “  “ “ | 21.290  “ “  “ “ |
|  | **III** | *Oncorhynchus mykiss* | FA281107 | EU481506 | V230308-5 (FJ362515) BV060408-52 (FJ362510) | [88]  “ “  “ “ | NOR “ “  “ “ | __ “ “  “ “ | __ “ “  “ “ |
|  | **IVa** | *Clupea harengus* | BC93-390  BC96-265-3 BC96-265-6 | KC117214  KC117217  KC117218 | __ | [79]  “ “  “ “ | Campbell R., BC, CAN  “ “  “ “ | 50.024  “ “  “ “ | -125.248  “ “  “ “ |
|  |  | *“ “* | ME03 | DQ401192 | __ | [5] | N. Pacific, USA | 47.622 | -122.638 |
|  |  | *C. harengus pallasii* | BC98-249  Campbell River248 BC 1993 | KC117219  U88051 | __ | [79]  [71] | Salt Spring Is., BC, CAN  Campbell R., BC, CAN | 48.815  50.024 | -123.508  -125.248 |
|  |  | *“ “* | BC00-LF | KC117220 | __ | [79] | Nanoose Harb., BC, CAN | 49.161 | -123.929 |
|  |  | *“ “* | BC00-397 | KC117221 | __ | “ “ | Hectate Strt., BC, CAN | 53.193 | -130.821 |
|  |  | *“ “* | BC02-47-21 | KC117226 | __ | “ “ | Stopper Is., Barkley Sound, BC, CAN | 48.983 | -125.353 |
|  |  | *“ “* | BC02-235-2 | KC117227 | __ | “ “ | Hardy Bay, BC, CAN | 50.712 | -127.491 |
|  |  | *“ “* | BC05-014-2  BC05-014-7 | KC117233  KC117234 | __ | “ “  “ “ | Clayoquot Sound, BC, CAN  “ “ | 49.250  “ “ | -126.000  “ “ |
|  |  | *“ “* | BC07-13-2 | KC117245 | __ | “ “ | Arrow Pass, BC, CAN | 48.450 | -123.330 |
|  |  | *“ “* | BC09-31-8  BC09-31-2 | KC117247  KC117248 | __ | “ “  “ “ | Port Hardy, BC, CAN  “ “ | 50.717  “ “ | -127.500  “ “ |
|  |  | *“ “* | WA-93 | DQ473303 | __ | [4] | Elliott Bay, WA, USA | 47.622 | -122.638 |
|  |  | *“ “* | U88052  AK93#1 | U88052  Z93430 | __ | [71]  [86] | Prince William Sound, AK, USA  “ “ | 61.622  “ “ | -146.863  “ “ |
|  |  | *“ “* | BC93372 | DQ401186 | __ | [5] | Prince Rupert Harb., BC, CAN | 54.301 | -130.332 |
|  |  | *“ “* | BC99010  BC99001 | DQ401194  DQ401195 | __  __ | “ “  “ “ | Gilford Is., BC, CAN  Beaver Cove, Telegraph Ck., BC, CAN | 50.717  50.532 | -126.369  -126.856 |
|  |  | *Cymatogaster aggregata* | BC-sp-02 | DQ473301 | __ | [4] | NW Vancouver Is., BC, CAN | 49.233 | -123.100 |
|  |  | *“ “* | BC02-41-14 | KC117225 | __ | [79] | Fair Harb., Kyuquot Sound, BC, CAN | 50.066 | -127.129 |
|  |  | *Gadus macro-cephalus* | AK’93  NA-6, NA-7  AK-90 | Z93429  Z93424-5  DQ473302 | __ | [86]  “ “  [4] | Prince William Sound, AK, USA “ “ “ “ | 61.622  “ “  “ “ | -146.863  “ “ “ “ |
|  |  | *“ “* | Clearwater WA 1991 | U88050 | __ | [71] | WA, USA | __ | __ |
|  |  | *Merluccius productus* | BC11-191 | KC117251 | __ | [79] | Bedwell Sound, BC, CAN | 50.124 | -127.129 |
|  |  | *“ “* | WA91 Clearwater | DQ401189 | __ | [5] | WA, USA | __ | __ |
|  |  | *Oncorhynchus kisuth* | Makah  Elok  NA-5  NA-8 | U28747 Z93421  Z93422  Z93423  Z93426 | __ | [71]  [86]  “ “  “ “  “ “ | Makah National Fish Hatchery, Neah Bay, WA, USA  Elokomin River, WA, USA  Bogachiel R., WA, USA  Clearwater R., WA, USA | 48.290  46.224  47.902  47.733 | -124.650  -123.330  -124.195  -124.015 |
|  |  | *O. mykiss* | RtGw11  RtGw10  RtGw5 | HQ687070  HQ687071  HQ687073 | RtGw8 (HQ687072) | Suebsing and Kim (unpub)  “ “  “ “  “ “ | S. KOR  “ “  “ “  “ “ | __  “ “  “ “  “ “ | __  “ “  “ “  “ “ |
|  |  | *O. tshawytscha* | BC301-1A  BC283-1B  BC313-1A | KC117235  KC117236  KC117237 | __ | [79]  “ “  “ “ | W. Coast Vancouver Is., BC, CAN  “ “  “ “ | 49.900  “ “  “ “ | -125.170  “ “  “ “ |
|  |  | *Paralichthys olivaceus* | AY167587 | AY167587 | __ | Kim and Park (unpub) | S. KOR | __ | __ |
|  |  | *“ “* | FWando08 | GU265811 | __ | Oh et al. (unpub) | S. KOR | __ | __ |
|  |  | *“ “* | KR-CJA | JQ651388 | KR-YGH (JQ651393) | Lee et al. (unpub) | Wando, KOR  Jeju, KOR | 34.389  33.430 | 126.702 126.546 |
|  |  | *“ “* | KRRV9822 | AB179621 | __ | Byon et al. (unpub) | S. of JPN, JPN | 32.000 | 132.000 |
|  |  | *“ “* | OfGn | HQ687076 | PhGn (HQ687075) PcGn (HQ687074) | Suebsing and Kim (unpub)  “ “  “ “ | S. KOR  “ “  “ “ | __  “ “  “ “ | __  “ “  “ “ |
|  |  | *“ “* | JP99Obama25  #25  JF00Ehi1 | DQ401191  AB060725  AB490792 | __ | [5]  Nishizawa et al. (unpub)  Ito et al. (unpub) | Obama coastal area of the Wakasa Bay, JPN  “ “  Ehime, JPN | 35.500  “ “  33.750 | 135.700  “ “  132.600 |
|  |  | *“ “* | KJ2008 | JF792424 | FYeosu05 (FJ811901)  FWando05 (FJ811900)  FJeju05 (FJ811902) FYG08 (GU265812) | Kim and Kim (unpub),  Kim et al. (unpub)  “ “  “ “  Oh et al. (unpub) | S. KOR  “ “  “ “  “ “  “ “ | __  “ “  “ “  “ “  “ “ | __  “ “  “ “  “ “  “ “ |
|  |  | *“ “* | GCVP-01  GCVP-03  GCVP-07  GCVP-09  GCVP-12  GCVP-18  GCVP-20  GCVP-27 | JQ952775  JQ952776  JQ952777  JQ952778  JQ952779  JQ952780  JQ952781  JQ952782 | __ | Han et al. (unpub)  “ “  “ “  “ “  “ “  “ “  “ “  “ “ | “ “  “ “  “ “  “ “  “ “  “ “  “ “  “ “ | “ “  “ “  “ “  “ “  “ “  “ “  “ “  “ “ | “ “  “ “  “ “  “ “  “ “  “ “  “ “  “ “ |
|  |  | *Salmo salar* | BC95-297 | KC117215 | __ | [79] | Campbell R., BC, CAN | 50.024 | -125.248 |
|  |  | *“ “* | BC02-03  BC07-286-10  BC07-15-7 | KC117223  KC117242  KC117244 | __ | “ “  “ “  “ “ | Arrow Pass, BC, CAN  “ “  “ “ | 48.450  “ “  “ “ | -123.330  “ “  “ “ |
|  |  | *“ “* | BC04-028-1  BC04-040  BC05-011 | KC117230  KC117231  KC117232 | __ | “ “  “ “  “ “ | Clayoquot Sound, BC, CAN  “ “  “ “ | 49.250  “ “  “ “ | -126.000  “ “  “ “ |
|  |  | *“ “* | BC06-37-2 | KC117241 | __ | “ “ | Bedwell Sound, BC, CAN | 50.124 | -127.129 |
|  |  | *“ “* | BC08-2816-1 | KC117246 | __ | “ “ | Port Hardy, BC, CAN | 50.717 | -127.50 |
|  |  | *“ “* | BC10-42-13 | KC117249 | __ | “ “ | Barkley Sound, BC, CAN | 48.849 | -125.386 |
|  |  | *Sardinops sagax* | BC02-28-6  BC02-41-9 | KC117222  KC117224 | __ | “ “  “ “ | Fair Harb., Kyuquot Sound, BC, CAN  “ “ | 50.066  “ “ | -127.129  “ “ |
|  |  | *“ “* | BC02-232-1 | KC117228 | __ | “ “ | Quatsino Sound, BC, CAN | 50.417 | -128.000 |
|  |  | *“ “* | BC02-229 | KC117229 | __ | “ “ | Hardy Bay, BC, CAN | __ | __ |
|  |  | *“ “* | BC06-089-1  BC-06-089-4 | KC117239  KC117240 | __ | “ “  “ “ | Rennell Sound, BC, CAN  “ “ | 53.417  “ “ | -132.750  “ “ |
|  |  | *“ “* | BC07-21-2 | KC117243 | __ | “ “ | Clayoquot Sound, BC, CAN | 49.250 | -126.000 |
|  |  | *“ “* | BC05-197 | KC117238 | __ | “ “ | Kitimat, BC, CAN | 54.063 | -128.578 |
|  |  | *“ “* | BC98250  BC99292 | DQ401187  DQ401188 | __ | [5]  “ “ | Salt Spring Is., BC, CAN  S. Vancouver Is., BC, CAN | 48.815  49.827 | -123.508  -125.620 |
|  |  | *“ “* | CMLs02 | DQ473300 | __ | [4] | Moss Landing, CA, USA | 36.804 | -121.786 |
|  |  | *“ “* | CMAs02 | DQ473299 | __ | “ “ | Malibu, CA, USA | 34.030 | -118.780 |
|  |  | *“ “* | CS01 | DQ473298 | __ | “ “ | Los Angeles, CA, USA | 34.052 | -118.242 |
|  |  | *“ “* | BC-s-99 | DQ473296 | __ | “ “ | Queen Charlotte, BC, CAN | 53.255 | -132.087 |
|  |  | *“ “* | Oe01 | DQ473297 | __ | “ “ | Sandy R., OR, USA | 45.401 | -122.228 |
|  | **IVb** | *Ambloplites rupestris* | i. TAVgr08-03  (vcG009) | HQ623441 | __ | [6] | N. Point Marina, L. MI, IL, USA | 42.499 | -90.689 |
|  |  | *Aplodinotus grunniens* | b. U13653-1  (vcG002) | HQ453209 | RG06 (EF564588) U13653-2  TAVgr06-02  TAVgr06-27  TAVgr08-04  TAVgr06-19  TAVgr06-03  TAVgr06-04  TAVgr06-31  TAVgr06-32  TAVgr06-33  TAVgr06-34  TAVgr06-35  TAVgr06-36  TAVgr06-39  TAVgr06-37  TAVgr06-38  TAVgr06-24  TAVgr06-20  TAVgr06-25  TAVgr06-21  TAVgr06-26  TAVgr06-23  TAVgr06-40  TAVgr06-22  TAVgr08-07  TAVgr07-08  TAVgr08-08  TAVgr08-02  TAVgr08-06  TAVgr08-05  TAVgr06-30  TAVgr06-01  TAVgr07-18  TAVgr07-19 | [25]  [6] “ “  “ “  “ “  “ “  “ “  “ “  “ “  “ “  “ “  “ “  “ “  “ “  “ “  “ “  “ “  “ “  “ “  “ “  “ “  “ “  “ “  “ “  “ “  “ “  “ “  “ “  “ “  “ “  “ “  “ “  “ “  “ “  “ “  “ “ | Bay of Quinte, L. Ont., CAN  St. Lawrence R., NY, USA  Dunkirk Harb., L. Erie, NY, USA  L. Huron, USA  L. Michigan, WI, USA  Fairhaven State Park, L. Ont., NY, USA  Oswego, L. Ont., NY, USA  Tibbetts Ck., L. Ont. USA  W. of Rochester, L. Ont., USA L. Skaneateles, NY, USA | 43.968    44.250  “ “  “ “  “ “  “ “  44.327  44.127  “ “  44.257  44.323  “ “  44.248  44.254  44.187  “ “  44.175  44.323  44.172  44.323  “ “  44.242  44.187  “ “  44.268  44.254  43.340  42.490  45.625  42.799  43.350  43.450  44.116  43.216  42.490 | -77.629  -76.000  “ “  “ “  “ “  “ “  -75.937  -76.333  “ “  -76.134  -76.014  “ “  -76.014  -76.014  -76.014  “ “  -76.964  -76.014  -76.247  -75.935  “ “  -76.098  -76.225  “ “  -76.014  -76.150  -75910  -79.338    -84.468  -87.760  -76.690  -76.510  -76.333  -77.633  -79.338 |
|  |  | *“ “* | l. *Drum2012 | KF928906 | __ | This study | Sandusky B., L. Erie, OH, USA | 41.453 | -82.726 |
|  |  | *Cyprinus carpio* | c. TAVgr07-12  (vcG003) | HQ623435 | TAVgr07-17  TAVgr07-13 | [6]  “ “  “ “ | Dunkirk Harb., L. Erie, NY, USA Little Salmon R., NY, USA  Skaneateles L., NY, USA | 42.490  43.459  42.950 | -79.338  -76.228  -76.230 |
|  |  | *“ “* | r. TAVgr11-02 (vcG028) | __ | vcG028 | [80] | Menomonee R. Canal, WI, USA | 43.030 | -87.915 |
|  |  | *Esox masquinongy* | a. MI03GL | GQ385941 | DQ401193  TAVgr05-01  0601FD  0602SB  0603BG  TAVgr06-48  TAVgr06-51  TAVgr06-52  TAVgr06-50  TAVgr06-47  TAVgr06-49  TAVgr09-03  TAVgr09-04  TAVgr09-10  TAVgr09-11  TAVgr07-09  TAVgr09-05  TAVgr06-07  TAVgr06-08  TAVgr06-09  TAVgr06-10  TAVgr06-11  TAVgr06-15  TAVgr07-21  TAVgr07-22  TAVgr08-09  TAVgr08-10  TAVgr08-11  TAVgr06-05  TAVgr06-06  TAVgr06-12  TAVgr06-13  TAVgr06-14  TAVgr06-17  TAVgr06-18  TAVgr06-46  TAVgr06-44  TAVgr06-43,  TAVgr06-45  TAVgr09-01  TAVgr09-02  TAVgr09-12  OMNR#5577  OMNR#5583  OMNR#5579  TAVgr06-28  TAVgr07-06  TAVgr07-07  TAVgr06-29  TAVgr10-01  TAVgr09-09  TAVgr07-02  TAVgr07-03  TAVgr07-14    TAVgr07-15  TAVgr07-16  TAVgr07-10  TAVgr07-11  TAVgr09-32    TAVgr09-33  TAVgr07-05  TAVgr09-13 | [40] [5]  [6] “ “  “ “  “ “  “ “  “ “  “ “  “ “  “ “  “ “  “ “  “ “  “ “  “ “  “ “  “ “  “ “  “ “  “ “  “ “  “ “  “ “  “ “  “ “  “ “  “ “  “ “  “ “  “ “  “ “  “ “  “ “  “ “  “ “  “ “  “ “  “ “  “ “  “ “  “ “  “ “  “ “  “ “  “ “  “ “  “ “  “ “  “ “  “ “  “ “  “ “  “ “  “ “  “ “  “ “  “ “  “ “  “ “  “ “  “ “ | L. St. Clair, MI,  USA  Dunkirk Harb., L. Erie, NY, USA L. Erie, OH, USA  L. Erie, OH, USA    Middle Basin, L. Erie, OH, USA  “ “  “ “  Sandusky Bay, L. Erie, OH, USA  “ “  W. L. Erie, OH,  USA  “ “  “ “  “ “  “ “  Cheboygan Bay, L. Huron, MI, USA  Swan R., L. Huron, MI, USA  Thunder Bay, L. Huron, MI, USA  “ “  L. Mich. MI, USA  “ “  Sturgeon Bay, L. Mich., WI, USA  Hamilton Harb.  L. Ont., ON, CAN  Thames R., L. ON, ON, CAN  Irondequoit Bay, L. Ont., NY, USA L. Ont., Rochester, NY, USA  “ “  Sodus Bay, L. Ont., NY, USA  Apostle Is., L. Super., WI, USA Baseline L., MI, USA  Budd L., MI, USA  “ “  Cayuga-Seneca Canal, NY, USA  “ “  “ “  Ransomville, NY, USA  “ “  Clear Fork Res., OH, USA  “ “  L. Winnebago, WI, USA  Oak Ck./Grant Pk., WI, USA | 42.390  “ “  __  __  __  42.343  “ “  “ “  “ “  42.634  “ “  42.631  “ “  42.615  42.475  42.490  __  41.755  “ “  “ “  “ “  “ “  “ “  41.801  “ “  41.769  41.769  41.823  41.474  “ “  41.492  “ “  “ “  “ “  “ “  45.718  45.502  45.050  “ “  “ “  43.600  44.860  43.295  “ “  42.328  43.200  43.233  “ “  42.257  47.085  42.427  44.015  “ “  42.910  “ “  “ “  43.240  “ “  40.716  “ “  44.028  42.926 | -82.911  “ “  __  __  __  -82.902  “ “  “ “  “ “  -82.777  “ “  -82.765  “ “  -82.757  -82.879  -79.338  __  -81.286  “ “  “ “  “ “  “ “  “ “  -81.356  “ “  -81.294  -81.353  -81.353  -82.703  “ “  -82.667  “ “  “ “  “ “  “ “  -84.374  -83.783  -83.200  “ “  “ “  -86.916  -87.393  -79.772  “ “  -82.472  -77.526  -76.650  “ “  -76.966  -90.641  -83.899  -84.788  “ “  -76.910  “ “  “ “  -79.920  “ “  -82.643  “ “  -88.421  -87.770 |
|  |  | *Lepomis gibbosus* | f. TAVgr07-04  (vcG006) | HQ623438 | __ | “ “ | Budd L. MI, USA | 44.015 | -84.788 |
|  |  | *L. macrochirus* | e. TAVgr07-01  ( vcG005) | HQ623437 | __ | “ “ | “ “ | “ “ | “ “ |
|  |  | *“ “* | g. TAVgr07-20  (vcG007) | HQ623439 | __ | “ “ | East Harb., L. Erie, OH, USA | 41.541 | -82.789 |
|  |  | *Micropterus dolomieu* | h. TAVgr07-24  (vcG008) | HQ623440 | __ | “ “ | Sturgeon Bay, L. Michigan, MI, USA | 44.884 | -87.388 |
|  |  | *M. salmoides* | k. *LMB2012 | KF928905 | __ | This study | Sandusky B., L. Erie, USA | 41.453 | -82.726 |
|  |  | *Morone chrysops* | d. TAVgr06-16  (vcG004) | HQ623436 | TAVgr08-01 | “ “  [6] | Western Basin, L. Erie, OH, USA  Clear Fork Res., OH, USA | 41.492  40.716 | -81.667  -82.643 |
|  |  | *Neogobius melanostomus* | t. Goby 1-5 | AB672615 | __ | [89] | Cape Vincent, L. Ontario NY, USA | 44.126 | -76.334 |
|  |  | *“ “* | o. vcG014 | __ | __ | [7] | Selkirk, St. Lawrence R., USA | 43.577 | -76.203 |
|  |  | *“ “* | p. TAVgr10-10  (vcG015) | __ | TAVgr10-08 | “ “  [6] | Cape Vincent, St. Lawrence R., USA  Selkirk Shores St. Pk., L. Ontario, USA | 44.185    43.577 | -76.224  -76.203 |
|  |  | *“ “* | q. vcG016 | __ | __ | [7] | Selkirk, St. Lawrence R., USA | “ “ | “ “ |
|  |  | *“ “* | s. GL2010-098 | KC117250 | __ | [79] | L. Simcoe, Ontario, CAN | 44.437 | -79.339 |
|  |  | *Perca flavescens* | j. TAVgr09-17  (vcG010) | HQ623442 | __ | [6] | L. Michigan, WI, USA | 43.039 | -87.802 |
|  |  | *Percopsis omiscomaycus* | m. TAVgr06-53  (vcG011) | HQ623443 | __ | “ “ | Central Basin, L. Erie, OH, USA | 41.755 | -81.286 |
|  |  | *“ “* | n. TAVgr10-02  (vcG013) | __ | __ | [80] | Hammond Bay, L. Michigan, MI, USA | 45.518 | -84.085 |
|  | **IVc** | *Fundulus heteroclitus* | CA-NB00-01 | EF079896 | __ | [90] | Ruisseau George Collette, near Bouctouche, NB, CAN | 46.450 | -64.682 |
|  |  | *Gasterosteus aculeatus* | CA-NB00-02 | HQ168405 | __ | “ “ | “ “ | “ “ | “ “ |
|  |  | *Morone saxatilis* | CA-NB04-01b | HQ453208 | __ | [6] | Miramichi Bay, Baie du Vin, NB, CAN | 47.163 | -64.574 |
|  |  | *“ “* | CA-NB02-01 | EF079897 | CA-NB04-01 (EF079898) | [90]  “ “ | “ “  Miramichi R., NB, CAN | “ “  47.004 | “ “  -65.541 |
|  |  | *S. trutta* | CA-NS04-01 | EF079899 | __ | “ “ | French R., NS, CAN | 45.576 | -62.425 |
| ***Nv*** | **I** | *Oncorhynchus kisutch* | Hededam | Z93412 | __ | [86] | Spjarup Hededamme, DNK | 56.648 | 9.271 |
|  | **II** | *Ciliata mustela* | DK-1p49 | DQ159193 | __ | [91] | Baltic S., DEU | 56.500 | 19.000 |
|  | **III** | *Oncorhynchus mykiss* | FA281107 | EU481506 | V230308-5 (FJ362515) KH230208-2 (FJ362513)  H050308-4 (FJ362512) BV060408-52 (FJ362510) | [88]  “ “ “ “  “ “ “ “ | NOR  “ “ “ “  “ “ “ “ | __  “ “  “ “   “ “ “ “ | __  “ “ “ “  “ “ “ “ |
|  | **IVa** | *Oncorhynchus kisutch* | Makah | U28745 | US Makah (DQ159204) | [92]  [91] | Makah National Fish Hatchery, Neah Bay, WA, USA | 48.290  __ | -124.650  __ |
|  |  | *“ “* | JF00Ehi1 | AB490792 | __ | Ito et al. (unpub) | Ehime, JPN | 33.750 | 132.600 |
|  |  | *Paralichthys olivaceus* | KRRV9822 | AB179621 | __ | Byon et al. (unpub) | JPN | __ | __ |
|  |  | *“ “* | KJ2008 | JF792424 | __ | Kim and Kim (unpub) | S. KOR | __ | __ |
|  |  | *“ “* | KR-CJA | JQ651389 | KR-YGH (JQ651394) | Lee et al. (unpub) | Wando, KOR  Jeju, KOR | 34.389  33.430 | 126.702 126.546 |
|  | **IVb** | *Ambloplites rupestris* | *TAVgr08-03^a^ | XXXXXX | __ | This study | N. Point Marina, L. MI, IL, USA | 42.499 | -90.689 |
|  |  | *Aplodinotus grunniens* | *Drum2012 | KF928917 | __ | “ “ | Sandusky B., L. Erie, USA | 41.453 | -82.726 |
|  |  | *Cyprinus carpio* | *TAVgr07-12^a^ | KF928908 | __ | “ “ | Dunkirk Harb., L. Erie, NY, USA | 42.490 | -79.338 |
|  |  | *Esox masquinongy* | MI03GL | GQ385941 | Goby 1-5 (AB672615) | [39]  [89] | L. St. Clair, MI,  USA  Cape Vincent, L. Ontario, NY, USA | 42.391  44.126 | -82.911  -76.334 |
|  |  | *Morone chrysops* | *TAvgr06-16^a^ | KF928909 | __ | This study | Western B., L. Erie, USA | 41.492 | -81.667 |
|  |  | *Micropterus dolomieu* | *TAVgr07-24^a^ | KF928913 | __ | “ “ | Sturgeon Bay, L. Michigan, MI, USA | 44.884 | -87.388 |
|  |  | *M. salmoides* | *LMB2012 | KF928916 | __ | “ “ | Sandusky B., L. Erie, USA | 41.453 | -82.726 |
|  |  | *Lepomis gibbosus* | *TAVgr07-04^a^ | KF928911 | __ | “ “ | Budd L., MI, USA | 44.015 | -84.788 |
|  |  | *L. macrochirus* | *TAVgr07-01^a^ | KF928910 | __ | “ “ | “ “ | “ “ | “ “ |
|  |  | *“ “* | *TAVgr07-20^a^ | KF928912 | __ | “ “ | East Harb., L. Erie, OH, USA | 41.541 | -82.789 |
|  |  | *Perca flavescens* | *TAVgr08-02^a^ | KF928907 | __ | “ “ | L. Michigan, near Milwaukee, USA | 42.799 | -87.761 |
|  |  | *“ “* | *TAVgr09-17^a^ | KF928915 | __ | “ “ | L. Michigan, WI, USA | 43.039 | -87.802 |
|  | **IVc** | *Gasterosteus aculeatus* | *CA-NB00-02^c^ | KF928918 | __ | “ “ | Ruisseau George Collette, near Bouctouche, NB, CAN | 46.450 | -64.682 |
| ***P*** | **I** | *Oncorhynchus mykiss* | Hededam | Z93412 | __ | [86] | Spjarup Hededamme, DNK | 56.648 | 9.271 |
|  | **II** | *Clupea harengus* | *FI-ka663-03^b^ | KF928925 | __ | This study | Archipelago S., FIN | 60.290 | 21.290 |
|  | **III** | *Oncorhynchus mykiss* | FA281107 | EU481506 | V230308-5 (FJ362515) KV010308-4 (FJ362514) BV060408-52 (FJ362510) | [88]  “ “ “ “  “ “ | NOR “ “  “ “  “ “ | __ “ “  “ “  “ “ | __ “ “  “ “  “ “ |
|  | **IVa** | *Onchorhychus kisutch* | Makah | U02630 | __ | [93] | Makah National Fish Hatchery, Neah Bay, WA, USA | 48.290 | -124.650 |
|  |  | *Paralichthys olivaceus* | KJ2008 | JF792424 | __ | Kim and Kim (unpub) | S. KOR | __ | __ |
|  |  | *“ ”* | Isolate #25 | AB060726 | __ | Nishizawa et al. (unpub) | Wakasa Bay, JPN | 35.200 | 134.240 |
|  |  | *“ ”* | KRRV9822 | AB179621 | __ | Byon et al. (unpub) | JPN | __ | __ |
|  | **IVb** | *Esox masquinongy* | MI03GL | GQ385941 | *Drum2012  *TAVgr07-12^a^  *TAVgr07-01^a^  *TAVgr07-04^a^  *TAVgr07-20^a^    *TAVgr07-24^a^  *TAVgr08-03^a^  *TAVgr09-17^a^ | [40]  This study  “ “  “ “  “ “  “ “  “ “  “ “  “ “ | L. St. Clair, MI,  USA  Sandusky B., L. Erie, USA  Dunkirk Harb., L. Erie, NY, USA  Budd L., MI, USA  “ “  East Harb., L. Erie, OH, USA  Sturgeon Bay, L. Michigan, MI, USA  N. Point Marina, L. MI, USA  L. Michigan, WI, USA | 42.391  41.453  42.490  44.015  “ “  41.541  44.884  42.499  43.039 | -82.911  -82.726  -79.338  -84.788  “ “  -82.789  -87.388  -90.689  -87.802 |
|  |  | *Micropterus salmoides* | *LMB2012 | KF928928 | __ | “ “ | Sandusky B., L. Erie, USA | 41.453 | -82.726 |
|  |  | *Morone chrysops* | *TAvgr06-16^a^ | KF928927 | __ | “ “ | Western B., L. Erie, USA | 41.492 | -82.667 |
|  |  | *Neogobius melanostomus* | t. Goby1-5 | AB672615 | __ | [89] | St. Lawrence R., near Cape Vincent, NY, USA | 44.383 | -75.868 |
|  |  | *Perca flavescens* | *TAVgr08-02^a^ | KF928926 | __ | This study | L. Michigan, near Milwaukee, USA | 42.799 | -87.761 |
|  | **IVc** | *Gasterosteus aculeatus* | *CA-NB00-02^c^ | KF928929 | __ | “ “ | Ruisseau George Collette, near Bouctouche, NB, CAN | 46.450 | -64.682 |
| ***M*** | **I** | *Onchorhychus mykiss* | Hededam | Z93412 | __ | [86] | Spjarup Hededamme, DNK | 56.648 | 9.271 |
|  | **III** | *Oncorhynchus mykiss* | FA281107 | EU481506 | V230308-5 (FJ362515) KV010308-4 (FJ362514) BV060408-52 (FJ362510) | [88]  “ “  “ “  “ “ | NOR  “ “  “ “  “ “ | __ “ “  “ “  “ “ | __ “ “  “ “  “ “ |
|  | **IVa** | *Onchorhychus kisutch* | Makah | U03503 | __ | [93] | Makah National Fish Hatchery, Neah Bay, WA, USA | 48.290 | -124.650 |
|  |  | *Paralichthys olivaceus* | KJ2008 | JF792424 | __ | Kim and Kim (unpub) | S. KOR | __ | __ |
|  |  | *“ “* | JF00Ehi1 | AB490792 | __ | Ito et al. (unpub) | Ehime Prefecture, JPN | 33.852 | 132.770 |
|  |  | *“ “* | KRRV9822 | AB179621 | __ | Byon et al. (unpub) | JPN | __ | __ |
|  |  | *“ “* | KR-CJA | JQ651387 | KR-YGH (JQ651392) | Lee et al. (unpub)  “ “ | S. KOR  “ “ | __  “ “ | __  “ “ |
|  | **IVb** | *Aplodinotus grunniens* | *Drum2012 | KF928923 | __ | This study | Sandusky Bay, L. Erie, USA | 41.453 | -82.726 |
|  |  | *Esox masquinongy* | MI03GL | GQ385941 | Goby 1-5 (AB672615)  *TAVgr07-12^a^  *TAvgr06-16^a^  *TAVgr07-01^a^  TAVgr07-04^a^  *TAVgr07-20^a^  *TAVgr08-03^a^ | [39]  [89]  This study  “ “  “ “  “ “  “ “  “ “ | L. St. Clair, MI,  USA  Cape Vincent, L. Ontario, NY, USA  Dunkirk Harb., L. Erie, NY, USA  Western B., L. Erie, USA  Budd L., MI, USA  “ “  East Harb., L. Erie, OH, USA  North Point Marina, L. MI, USA | 42.391  44.126  42.490  41.492  44.015  “ “  41.541  42.499 | -82.911  -76.334  -79.338  -81.667  -84.788  “ “  -82.789  -90.689  -90.689 |
|  |  | *Micropterus dolomieu* | *TAVgr07-24^a^ | KF928920 | __ | “ “ | Sturgeon Bay, L. Mich., MI, USA | 44.884 | -87.388 |
|  |  | *M. salmoides* | *LMB2012 | KF928922 | __ | “ “ | Sandusky Bay, L. Erie, USA | 41.453 | -82.726 |
|  |  | *Perca flavescens* | *TAVgr08-02^a^ | KF928919 | __ | “ “ | L. Mich., near Milwaukee, USA | 42.799 | -87.761 |
|  |  | *“ “* | *TAVgr09-17^a^ | KF928921 | __ | “ “ | L. Mich., WI, USA | 43.039 | -87.802 |
|  | **IVc** | *Gasterosteus aculeatus* | *CA-NB00-02^c^ | KF928924 | __ | “ “ | Ruisseau George Collette, near Bouctouche, NB, CAN | 46.450 | -64.682 |

^a^ Isolates from Western Fisheries Research Center, USGS, Seattle, WA, USA.

^b^ Isolates from Finnish Food Safety Authority, Evira, Finland.

^c^ Isolates from Fisheries and Oceans Canada, Pacific Biological Station, BC, Canada.

**Supplementary Table C**

(a) *G*-gene

| Haplotype and Isolate | GenBank  Accession No. | | Nucleotide Position - GenBank (and in reference to M103GL) and Codon Position | | | | | | | | |
| --- | --- | --- | --- | --- | --- | --- | --- | --- | --- | --- | --- |
|  |  | | 182 (3590) | 192 (3600) | 346 (3754) | 347 (3755) | 406 (3814) | 421 (3829) | 423 (3831) | 464 (3872) | 476 (3884) |
|  |  | | 3 | 1 | 2 | 3 | 2 | 2 | 1 | 3 | 3 |
|  |  | |  |  |  |  |  |  |  |  |  |
| a. MI03GL | | GQ385941 | A  (gly) | A  (ile) | C  (ala) | A  (ala) | A  (lys) | G  (arg) | A  (lys) | C  (asp) | C  (tyr) |
| b. TAVgr08-02   (vcG002) | | EF564588 | . | . | . | . | . | . | . | . | . |
| c. TAVgr07-12   (vcG003) | | HQ623435 | . | . | . | . | . | . | . | . | . |
| d. TAvgr06-16   (vcG004) | | HQ623436 | . | . | . | . | . | . | . | . | . |
| e. TAVgr07-01   (vcG005) | | HQ623437 | G | . | . | . | . | . | . | . | . |
| f. TAVgr07-04   (vcG006) | | HQ623438 | . | . | A^+^  (glu) | . | . | . | . | . | . |
| g. TAVgr07-20   (vcG007) | | HQ623439 | . | . | . | . | . | . | . | . | . |
| h. TAVgr07-24   (vcG008) | | HQ623440 | . | G^+^ (val) | . | . | . | A^+^  (lys) | . | . | . |
| i. TAvgr08-03   (vcG009) | | HQ623441 | . | . | . | . | G^+^  (arg) | . | G^+^ (glu) | . | . |
| j. TAVgr09-17   (vcG010) | | HQ623442 | . | . | . | G | . | . | . | . | . |
| k. LMB2012* | | KF928905 | . | . | . | . | . | . | . | T | . |
| l. Drum2012* | | KF928906 | . | . | . | . | . | A^+^  (lys) | . | T | T |

| Haplotype and Isolate | GenBank  Accession No. | Nucleotide Position - GenBank (and in reference to M103GL) and Codon Position | | | | |
| --- | --- | --- | --- | --- | --- | --- |
|  |  | 521  (3929) | 561  (3969) | 588  (3996) | 590  (3998) | 599  (4007) |
|  |  | 3 | 1 | 1 | 3 | 3 |
|  |  |  |  |  |  |  |
| a. MI03GL | GQ385941 | G  (leu) | T  (ser) | A  (ser) | T  (ser) | C  (gly) |
| b. TAVgr08-02   (vcG002) | EF564588 | . | . | . | . | G |
| c. TAVgr07-12   (vcG003) | HQ623435 | A | . | . | . | . |
| d. TAvgr06-16   (vcG004) | HQ623436 | . | . | C^+^  (arg) | . | . |
| e. TAVgr07-01   (vcG005) | HQ623437 | . | . | . | . | . |
| f. TAVgr07-04   (vcG006) | HQ623438 | . | . | . | . | . |
| g. TAVgr07-20   (vcG007) | HQ623439 | . | . | . | A^+^  (arg) | . |
| h. TAVgr07-24   (vcG008) | HQ623440 | . | . | . | . | . |
| i. TAvgr08-03   (vcG009) | HQ623441 | . | C^+^  (pro) | . | . | G |
| j. TAVgr09-17   (vcG010) | HQ623442 | . | . | . | . | . |
| k. LMB2012* | KF928905 | . | . | . | . | . |
| l. Drum2012* | KF928906 | . | . | . | . | . |

(b) *Nv*-gene

| Haplotype  and Isolate | GenBank  Accession No. | Nucleotide Position - GenBank (and in reference to M103GL) and Codon Position | | | | | | | | | | | | |
| --- | --- | --- | --- | --- | --- | --- | --- | --- | --- | --- | --- | --- | --- | --- |
|  |  | 8 (4587) | 13 (4592) | 34 (4613) | 72 (4651) | 79 (4658) | 87 (4666) | 111 (4690) | 120 (4699) | 127 (4706) | 132 (4711) | 133 (4712) | 139 (4718) | 153 (4732) |
|  |  | 2 | 1 | 1 | 3 | 1 | 3 | 3 | 3 | 1 | 3 | 1 | 1 | 3 |
|  |  |  |  |  |  |  |  |  |  |  |  |  |  |  |
| a. MI03GL | GQ385941 | T  (ile) | C  (pro) | T  (phe) | A  (arg) | T  (phe) | A  (pro) | C  (ile) | A  (ser) | T  (ser) | T  (ala) | A  (thr) | T  (phe) | T (thr) |
| b. TAVgr08-02* | KF928907 | C^+^  (thr) | T^+^  (ser) | . | . | . | . | . | . | . | . | . | . | . |
| c. TAVgr07-12* | KF928908 | C^+^  (thr) | T^+^  (ser) | . | . | . | . | . | . | C^+^  (pro) | C | . | C^+^  (leu) | . |
| d. TAvgr06-16* | KF928909 | C^+^  (thr) | T^+^  (ser) | . | G | . | . | . | C | . | . | . | . | . |
| e. TAVgr07-01* | KF928910 | C^+^  (thr) | T^+^  (ser) | C^+^  (leu) | . | . | . | . | . | . | . | . | . | . |
| f. TAVgr07-04* | KF928911 | C^+^  (thr) | T^+^  (ser) | . | . | . | . | . | . | . | . | . | . | . |
| g. TAVgr07-20* | KF928912 | C^+^  (thr) | T^+^  (ser) | . | . | . | G | . | . | . | . | . | . | . |
| h. TAVgr07-24* | KF928913 | C^+^  (thr) | T^+^  (ser) | . | . | . | . | . | C | . | . | . | . | C |
| i. TAvgr08-03* | KF928914 | C^+^  (thr) | T^+^  (ser) | . | . | G^+^  (val) | . | . | . | . | . | G^+^  (ala) | . | . |
| j. TAVgr09-17* | KF928915 | C^+^  (thr) | T^+^  (ser) | . | . | . | . | . | . | . | . | . | . | . |
| k. LMB2012* | KF928916 | . | . | C^+^  (leu) | . | . | . | . | . | . | . | . | . | . |
| l. Drum2012* | KF928917 | . | . | C^+^  (leu) | . | . | . | T | . | . | . | . | . | . |

| Haplotype and Isolate | Nucleotide Position - GenBank (and in reference to M103GL) and Codon Position | | | | | | | | | | | | | |
| --- | --- | --- | --- | --- | --- | --- | --- | --- | --- | --- | --- | --- | --- | --- |
|  | 157 (4736) | 159 (4738) | 166 (4745) | 167 (4746) | 174 (4753) | 176 (4755) | 179 (4758) | 194 (4773) | 199 (4778) | 210 (4789) | 213 (4792) | 218 (4797) | 224 (4803) | 233 (4812) |
|  | 1 | 3 | 1 | 2 | 3 | 2 | 2 | 2 | 1 | 3 | 3 | 2 | 2 | 2 |
|  |  |  |  |  |  |  |  |  |  |  |  |  |  |  |
| a. MI03GL | T  (ser) | T  (ser) | T  (leu) | T  (leu) | T  (asp) | T  (leu) | G  (arg) | T  (leu) | T  (tyr) | G  (val) | T  (leu) | T  (leu) | T  (ile) | T  (leu) |
| b. TAVgr08-02* | . | C | C | C^+^  (ser) | C | C^+^  (pro) | . | . | . | . | . | . | . | . |
| c. TAVgr07-12* | C | . | . | . | . | . | . | C^+^  (pro) | C^+^  (his) | . | C | C^+^  (pro) | C^+^  (thr) | C^+^  (pro) |
| d. TAvgr06-16* | . | . | . | . | . | . | . | . | . | . | . | . | . | . |
| e. TAVgr07-01* | . | . | . | . | . | . | . | . | . | A | . | . | . | . |
| f. TAVgr07-04* | . | . | . | . | . | . | . | . | . | . | . | . | . | . |
| g. TAVgr07-20* | . | . | . | . | . | . | . | . | . | . | . | . | . | . |
| h. TAVgr07-24* | . | . | . | . | . | . | A^+^  (lys) | . | . | . | . | . | . | . |
| i. TAvgr08-03* | . | . | . | . | . | . | . | . | . | . | . | . | . | . |
| j. TAVgr09-17* | . | . | . | . | . | . | . | . | . | . | . | . | . | . |
| k. LMB2012* | . | . | . | . | . | . | . | . | . | . | . | . | . | . |
| l. Drum2012* | . | . | . | . | . | . | . | . | . | . | . | . | . | . |

| Haplotype and Isolate | Nucleotide Position - GenBank (and in reference to M103GL) and Codon Position | | | | | | | | | | | | | |
| --- | --- | --- | --- | --- | --- | --- | --- | --- | --- | --- | --- | --- | --- | --- |
|  | 243 (4822) | 247 (4826) | 251 (4830) | 254 (4833) | 270 (4849) | 276 (4855) | 279 (4858) | 288 (4867) | 303 (4882) | 310 (4889) | 313 (4892) | 323 (4902) | 324 (4903) | 332 (4911) |
|  | 3 | 1 | 2 | 2 | 3 | 3 | 3 | 3 | 3 | 1 | 1 | 2 | 3 | 2 |
|  |  |  |  |  |  |  |  |  |  |  |  |  |  |  |
| a. MI03GL | T  (thr) | T  (tyr) | T  (ile) | T  (leu) | T  (ser) | T  (pro) | T  (ala) | T  (gly) | T  (pro) | T  (phe) | A  (ile) | T  (leu) | T  (leu) | T  (met) |
| b. TAVgr08-02* | . | . | C^+^  (thr) | C^+^  (pro) | . | C | . | . | . | . | . | C^+^  (pro) | . | . |
| c. TAVgr07-12* | C | C^+^  (his) | . | C^+^  (pro) | C | . | C | C | C | C^+^  (leu) | . | C^+^  (pro) | C^+^  (pro) | C^+^  (thr) |
| d. TAvgr06-16* | . | . | . | . | . | . | . | . | . | . | . | . | . | . |
| e. TAVgr07-01* | . | . | . | . | . | . | . | . | . | . | . | . | . | . |
| f. TAVgr07-04* | . | . | . | . | . | . | . | . | . | . | G^+^  (val) | . | . | . |
| g. TAVgr07-20* | . | . | . | . | . | . | . | . | . | . | . | . | . | . |
| h. TAVgr07-24* | . | . | . | . | . | . | . | . | . | . | . | C^+^  (pro) | . | . |
| i. TAvgr08-03* | . | . | . | . | . | . | . | . | . | . | . | . | . | . |
| j. TAVgr09-17* | . | . | . | . | . | . | . | . | . | . | . | . | . | . |
| k. LMB2012* | . | . | . | . | . | . | . | . | . | . | . | . | . | . |
| l. Drum2012* | . | . | . | . | . | . | . | . | . | . | . | . | . | . |

| Haplotype and Isolate | Nucleotide Position - GenBank (and in reference to M103GL) and Codon Position | | | | | | |
| --- | --- | --- | --- | --- | --- | --- | --- |
|  | 337 (4916) | 341 (4920) | 351 (4930) | 354 (4933) | 357 (4936) | 363 (4942) | 364 (4943) |
|  | 1 | 2 | 3 | 3 | 3 | 3 | 1 |
|  |  |  |  |  |  |  |  |
| a. MI03GL | A  (ile) | T  (leu) | C  (gly) | T  (ser) | G  (glu) | T  (pro) | T  (ser) |
| b. TAVgr08-02* | . | C^+^  (ser) | . | C | A | C | C^+^  (pro) |
| c. TAVgr07-12* | . | C^+^  (ser) | . | C | A | C | C^+^  (pro) |
| d. TAvgr06-16* | . | . | . | C | A | C | C^+^  (pro) |
| e. TAVgr07-01* | . | . | . | C | A | C | C^+^  (pro) |
| f. TAVgr07-04* | . | . | . | C | A | C | C^+^  (pro) |
| g. TAVgr07-20* | . | . | . | C | A | C | C^+^  (pro) |
| h. TAVgr07-24* | . | . | . | C | A | C | C^+^  (pro) |
| i. TAvgr08-03* | . | . | . | C | A | C | C^+^  (pro) |
| j. TAVgr09-17* | G^+^  (val) | . | . | C | A | C | C^+^  (pro) |
| k. LMB2012* | . | . | . | . | . | . | . |
| l. Drum2012* | . | . | A | . | . | . | . |

(c) *P*-gene

| Haplotype  and Isolate | GenBank  Accession No. | Nucleotide position in reference to aligned GenBank sequence (position in MI03GL complete genome sequence) | | | | | |
| --- | --- | --- | --- | --- | --- | --- | --- |
|  |  | 29 (1578) | 231 (1782) | 470 (2019) | 488 (2037) | 495 (2044) | 525 (2074) |
|  |  | 3 | 3 | 3 | 3 | 1 | 1 |
|  |  |  |  |  |  |  |  |
| a. MI03GL | GQ385941 | C  (asn) | T  (thr) | G  (lys) | C  (pro) | C  (leu) | C  (leu) |
| b. TAVgr08-02* | KF928926 | . | . | T^+^  (asn) | T | A^+^  (ile) | T^+^  (phe) |
| d. TAvgr06-16* | KF928927 | T | . | . | . | . | . |
| k. LMB2012* | KF928928 | . | C | . | . | . | . |

(d) *M*-gene

| Haplotype  And Isolate | | GenBank  Accession No. | Nucleotide Position in Reference to Aligned GenBank Sequence (Position in MI03GL Complete Genome Sequence) | | | | |
| --- | --- | --- | --- | --- | --- | --- | --- |
|  | |  | 111 (2436) | 153 (2478) | 329 (2654) | 372 (2697) | 543 (2868) |
|  |  | | 3 | 3 | 2 | 3 | 3 |
|  |  | |  |  |  |  |  |
| a. MI03GL | | GQ385941 | G  (met) | T  (his) | A  (lys) | T  (gly) | A  (gly) |
| b. TAVgr08-02* | | KF928919 | . | . | G^+^  (arg) | . | . |
| h. TAVgr07-24* | | KF928920 | T^+^  (ile) | . | . | . | . |
| j. TAVgr09-17* | | KF928921 | . | . | . | . | G |
| k. LMB2012* | | KF928922 | . | . | . | C | . |
| l. Drum2012* | | KF928923 | . | C | . | C | . |

**Supplementary Table D**

|  | Nucleotide Position and *Gene (in Italics)* | | | | | | | | | | | | | | | | | |
| --- | --- | --- | --- | --- | --- | --- | --- | --- | --- | --- | --- | --- | --- | --- | --- | --- | --- | --- |
| Isolate and  Haplotype | 1578 | 1782 | 2019 | 2037 | 2044 | 2074 | 2436 | 2478 | 2654 | 2697 | 2868 | 3590 | 3600 | 3754 | 3755 | 3814 | 3829 | 3831 |
|  | *P* | *P* | *P* | *P* | *P* | *P* | *M* | *M* | *M* | *M* | *M* | *G* | *G* | *G* | *G* | *G* | *G* | *G* |
| I | C | T | G | C | C | C | G | C | A | T | A | A | G | C | A | G | A | A |
| Hededam |  |  |  |  |  |  |  |  |  |  |  |  |  |  |  |  |  |  |
| III |  |  |  |  |  |  |  |  |  |  |  |  |  |  |  |  |  |  |
| FA281107 | C | T | G | T | C | C | G | C | A | C | A | A | G | C | A | C | A | A |
| IVa |  |  |  |  |  |  |  |  |  |  |  |  |  |  |  |  |  |  |
| Makah | C | T | G | C | C | C | G | T | A | T | A | A | A | C | A | A | A | A |
| JF00Ehi1 | C | T | G | C | C | C | G | T | A | T | A | A | A | C | A | A | A | A |
| KJ2008 | C | T | G | C | C | C | G | T | A | T | A | A | A | C | A | A | A | A |
| IVb |  |  |  |  |  |  |  |  |  |  |  |  |  |  |  |  |  |  |
| a | C | T | G | C | C | C | G | T | A | T | A | A | A | C | A | A | G | A |
| b | C | T | T | T | A | T | G | T | G | T | A | A | A | C | A | A | G | A |
| c | C | T | G | C | C | C | G | T | A | T | A | A | A | C | A | A | G | A |
| d | T | T | G | C | C | C | G | T | A | T | A | A | A | C | A | A | G | A |
| e | C | T | G | C | C | C | G | T | A | T | A | G | A | C | A | A | G | A |
| f | C | T | G | C | C | C | G | T | A | T | A | A | A | A | A | A | G | A |
| g | C | T | G | C | C | C | G | T | A | T | A | A | A | C | A | A | G | A |
| h | C | T | G | C | C | C | T | T | A | T | A | A | G | C | A | A | A | A |
| i | C | T | G | C | C | C | G | T | A | T | A | A | A | C | A | G | G | A |
| j | C | T | G | C | C | C | G | T | A | T | G | A | A | C | G | A | G | G |
| k | C | C | G | C | C | C | G | C | A | C | A | A | A | C | A | A | G | A |
| l | C | T | G | C | C | C | G | T | A | C | A | A | A | C | A | A | A | A |
| IVc |  |  |  |  |  |  |  |  |  |  |  |  |  |  |  |  |  |  |
| CA-NB00-02 | C | T | G | C | C | C | G | T | A | T | A | A | A | C | A | A | G | A |

|  | Nucleotide Position and *Gene (in Italics)* | | | | | | | | | | | | | | | | | |
| --- | --- | --- | --- | --- | --- | --- | --- | --- | --- | --- | --- | --- | --- | --- | --- | --- | --- | --- |
| Isolate and  Haplotype | 3872*G* | 3884*G* | 3929*G* | 3969*G* | 3996*G* | 3998*G* | 4007  *G* | 4586*Nv* | 4587  *Nv* | 4592  *Nv* | 4613  *Nv* | 4651  *Nv* | 4658  *Nv* | 4666  *Nv* | 4690  *Nv* | 4699  *Nv* | 4706  *Nv* | 4711  *Nv* |
| I |  |  |  |  |  |  |  |  |  |  |  |  |  |  |  |  |  |  |
| Hededam | C | T | A | T | A | C | A | A | C | C | T | A | T | A | T | G | T | C |
| III |  |  |  |  |  |  |  |  |  |  |  |  |  |  |  |  |  |  |
| FA281107 | C | T | A | T | A | C | A | A | C | C | T | G | T | A | C | G | T | C |
| IVa |  |  |  |  |  |  |  |  |  |  |  |  |  |  |  |  |  |  |
| Makah | C | T | G | C | A | T | A | A | C | T | T | A | T | A | C | A | T | T |
| JF00Ehi1 | C | T | G | C | A | T | G | A | C | T | T | A | T | A | C | A | T | T |
| KJ2008 | C | T | G | C | A | T | G | A | C | T | T | A | T | A | C | A | T | T |
| IVb |  |  |  |  |  |  |  |  |  |  |  |  |  |  |  |  |  |  |
| a | C | C | G | T | A | T | C | A | T | C | T | A | T | A | C | A | T | T |
| b | C | C | G | T | A | T | G | A | C | T | T | A | T | A | C | A | T | T |
| c | C | C | A | T | A | T | C | A | C | T | T | A | T | A | C | A | C | C |
| d | C | C | G | T | C | T | C | A | C | T | T | G | T | A | C | C | T | T |
| e | C | C | G | T | A | T | C | A | C | T | C | A | T | A | C | A | T | T |
| f | C | C | G | T | A | T | C | A | C | T | T | A | T | A | C | A | T | T |
| g | C | C | G | T | A | A | C | C | A | T | T | A | T | G | C | A | T | T |
| h | C | C | G | T | A | T | C | A | C | T | T | A | T | A | C | C | T | T |
| i | C | C | G | C | A | T | G | A | C | T | T | A | T | A | C | A | T | T |
| j | C | C | G | T | A | T | C | A | C | T | T | A | G | A | C | A | T | T |
| k | T | C | G | T | A | T | C | A | T | C | C | A | T | A | C | A | T | T |
| l | T | T | G | T | A | T | C | A | T | C | C | A | T | A | T | A | T | T |
| IVc |  |  |  |  |  |  |  |  |  |  |  |  |  |  |  |  |  |  |
| CA-NB00-02 | C | C | G | T | A | T | G | G | C | C | T | A | T | A | C | A | T | T |

|  | Nucleotide Position and *Gene (in Italics)* | | | | | | | | | | | | | | | | | |
| --- | --- | --- | --- | --- | --- | --- | --- | --- | --- | --- | --- | --- | --- | --- | --- | --- | --- | --- |
| Isolate and  Haplotype | 4712 | 4718 | 4732 | 4736 | 4738 | 4745 | 4746 | 4753 | 4755 | 4758 | 4773 | 4778 | 4789 | 4792 | 4797 | 4803 | 4812 | 4822 |
|  | *Nv* | *Nv* | *Nv* | *Nv* | *Nv* | *Nv* | *Nv* | *Nv* | *Nv* | *Nv* | *Nv* | *Nv* | *Nv* | *Nv* | *Nv* | *Nv* | *Nv* | *Nv* |
| I |  |  |  |  |  |  |  |  |  |  |  |  |  |  |  |  |  |  |
| Hededam | A | T | C | C | C | C | T | T | T | G | T | T | G | T | T | T | T | C |
| III |  |  |  |  |  |  |  |  |  |  |  |  |  |  |  |  |  |  |
| FA281107 | A | T | T | C | C | T | T | C | T | G | T | T | A | C | T | T | T | C |
| IVa |  |  |  |  |  |  |  |  |  |  |  |  |  |  |  |  |  |  |
| Makah | A | T | T | T | T | T | T | T | T | G | T | T | G | T | T | T | T | T |
| JF00Ehi1 | A | T | T | T | T | T | C | T | T | G | T | T | G | T | T | T | T | T |
| KJ2008 | A | T | T | T | T | T | C | T | T | G | T | T | G | T | T | T | T | T |
| IVb |  |  |  |  |  |  |  |  |  |  |  |  |  |  |  |  |  |  |
| a | A | T | T | T | T | T | T | T | T | G | T | T | G | T | T | T | T | T |
| b | A | T | T | T | C | C | C | C | C | G | T | T | G | T | T | T | T | T |
| c | A | C | T | C | T | T | T | T | T | G | C | C | G | C | C | C | C | C |
| d | A | T | T | T | T | T | T | T | T | G | T | T | G | T | T | T | T | T |
| e | A | T | T | T | T | T | T | T | T | G | T | T | A | T | T | T | T | T |
| f | A | T | T | T | T | T | T | T | T | G | T | T | G | T | T | T | T | T |
| g | A | T | T | T | T | T | T | T | T | G | T | T | G | T | T | T | T | T |
| h | A | T | C | T | T | T | T | T | T | A | T | T | G | T | T | T | T | T |
| i | G | T | T | T | T | T | T | T | T | G | T | T | G | T | T | T | T | T |
| j | A | T | T | T | T | T | T | T | T | G | T | T | G | T | T | T | T | T |
| k | A | T | T | T | T | T | T | T | T | G | T | T | G | T | T | T | T | T |
| l | A | T | T | T | T | T | T | T | T | G | T | T | G | T | T | T | T | T |
| IVc |  |  |  |  |  |  |  |  |  |  |  |  |  |  |  |  |  |  |
| CA-NB00-02 | A | T | T | T | T | T | T | T | T | G | T | T | G | T | T | T | T | T |

|  | Nucleotide Position and *Gene (in Italics)* | | | | | | | | | | | | | | | | | |
| --- | --- | --- | --- | --- | --- | --- | --- | --- | --- | --- | --- | --- | --- | --- | --- | --- | --- | --- |
| Isolate and  Haplotype | 4826 | 4830 | 4833 | 4849 | 4855 | 4858 | 4867 | 4882 | 4889 | 4892 | 4902 | 4903 | 4911 | 4916 | 4920 | 4930 | 4933 | 4936 |
|  | *Nv* | *Nv* | *Nv* | *Nv* | *Nv* | *Nv* | *Nv* | *Nv* | *Nv* | *Nv* | *Nv* | *Nv* | *Nv* | *Nv* | *Nv* | *Nv* | *Nv* | *Nv* |
|  |  |  |  |  |  |  |  |  |  |  |  |  |  |  |  |  |  |  |
| I |  |  |  |  |  |  |  |  |  |  |  |  |  |  |  |  |  |  |
| Hededam | T | T | T | T | C | C | T | T | A | A | A | T | T | A | T | C | T | G |
| III |  |  |  |  |  |  |  |  |  |  |  |  |  |  |  |  |  |  |
| FA281107 | T | T | T | C | C | C | C | T | A | A | G | T | T | G | T | C | C | G |
| IVa |  |  |  |  |  |  |  |  |  |  |  |  |  |  |  |  |  |  |
| Makah | T | T | T | C | T | T | T | T | T | A | T | T | T | A | T | C | C | G |
| JF00Ehi1 | T | T | T | C | T | T | T | T | T | A | T | T | T | A | T | C | C | A |
| KJ2008 | T | T | T | C | T | T | T | T | T | A | T | T | T | A | T | C | C | A |
| IVb |  |  |  |  |  |  |  |  |  |  |  |  |  |  |  |  |  |  |
| a | T | T | T | T | T | T | T | T | T | A | T | T | T | A | T | C | T | G |
| b | T | C | C | T | C | T | T | T | T | A | C | T | T | A | C | C | C | A |
| c | C | T | C | C | T | C | C | C | C | A | C | C | C | A | C | C | C | A |
| d | T | T | T | T | T | T | T | T | T | A | T | T | T | A | T | C | C | A |
| e | T | T | T | T | T | T | T | T | T | A | T | T | T | A | T | C | C | A |
| f | T | T | T | T | T | T | T | T | T | G | T | T | T | A | T | C | C | A |
| g | T | T | T | T | T | T | T | T | T | A | T | T | T | A | T | C | C | A |
| h | T | T | T | T | T | T | T | T | T | A | C | T | T | A | T | C | C | A |
| i | T | T | T | T | T | T | T | T | T | A | T | T | T | A | T | C | C | A |
| j | T | T | T | T | T | T | T | T | T | A | T | T | T | G | T | C | C | A |
| k | T | T | T | T | T | T | T | T | T | A | T | T | T | A | T | C | T | G |
| l | T | T | T | T | T | T | T | T | T | A | T | T | T | A | T | A | T | G |
| IVc |  |  |  |  |  |  |  |  |  |  |  |  |  |  |  |  |  |  |
| CA-NB00-02 | T | T | T | T | T | C | C | T | T | A | T | T | T | A | T | C | T | G |

|  | Nucleotide Position and *Gene (in Italics)* | |
| --- | --- | --- |
| Isolate and  Haplotype | 4943 | 4944 |
|  | *Nv* | *Nv* |
|  |  |  |
| I |  |  |
| Hededam | T | C |
| III |  |  |
| FA281107 | T | C |
| IVa |  |  |
| Makah | C | C |
| JF00Ehi1 | C | C |
| KJ2008 | C | C |
| IVb |  |  |
| a | T | T |
| b | C | C |
| c | C | C |
| d | C | C |
| e | C | C |
| f | C | C |
| g | C | C |
| h | C | C |
| i | C | C |
| j | C | C |
| k | T | T |
| l | T | T |
| IVc |  |  |
| CA-NB00-02 | T | C |

**Supplementary Figure A**
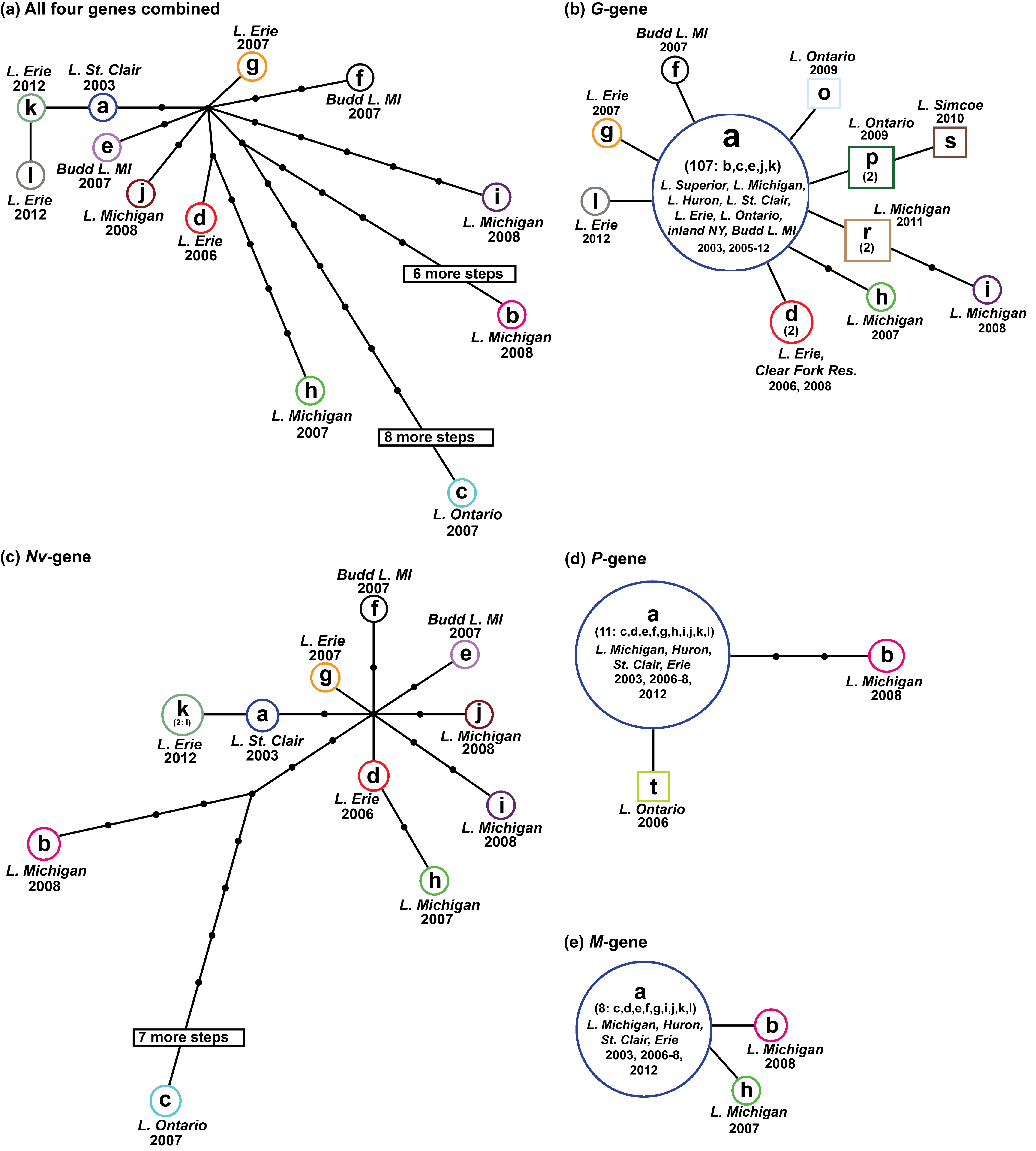


**Supplementary Figure B**


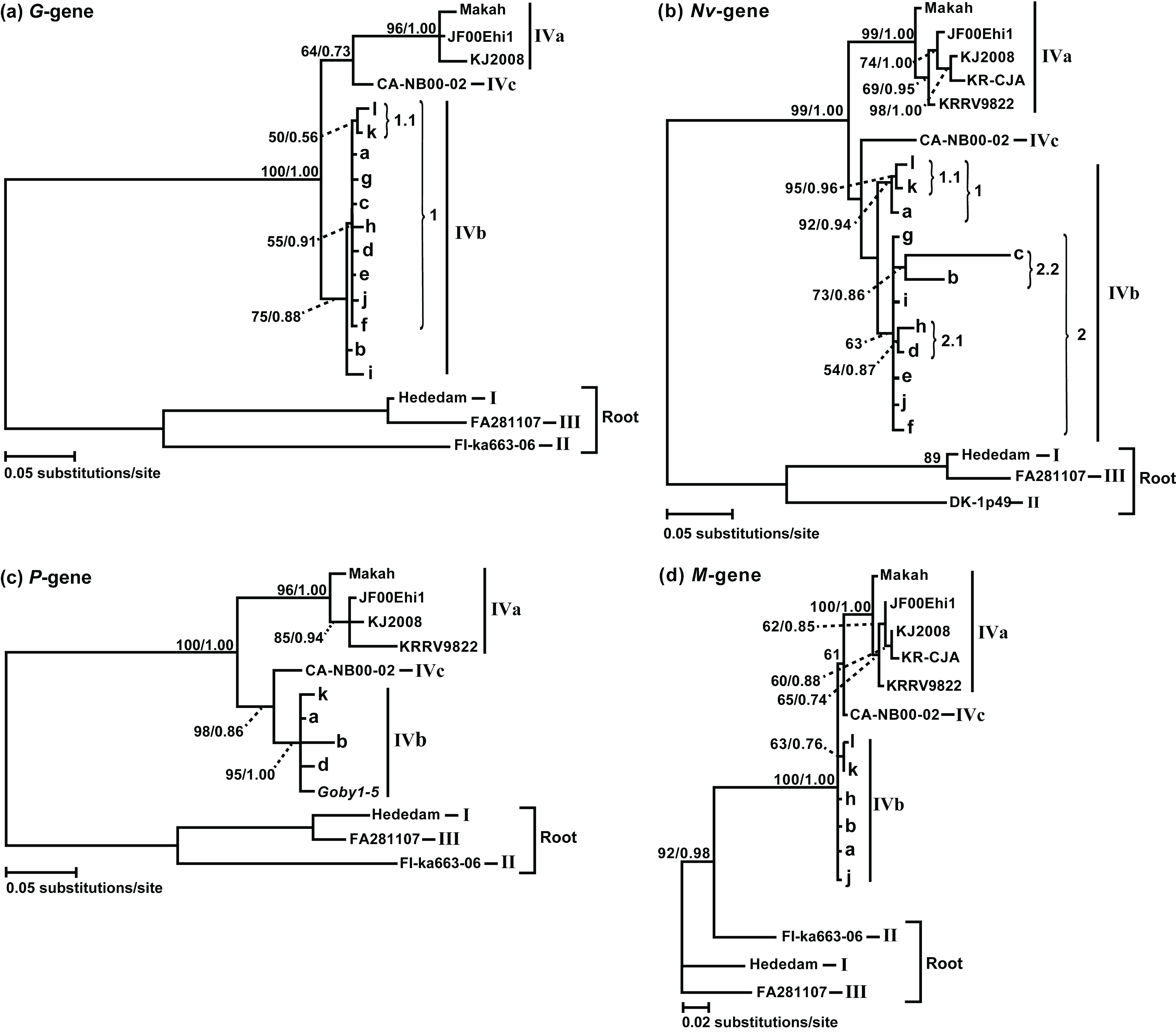


Additional References

1. Stone DM, Way K, Dixon PF. Nucleotide sequence of the glycoprotein gene of Viral Hemorrhagic Septicaemia (VHS) viruses from different geographical areas: A link between VHS in farmed fish species and viruses isolated from North Sea cod (*Gadus morhua L*). J Gen Virol. 1997;78: 1319–1326.
2. Gadd T, Jakava-Viljanen M, Tapiovaara H, Koski P, Sihvonen L. Epidemiological aspects of Viral Haemorrhagic Septicaemia virus genotype II isolated from Baltic herring, *Clupea harengus membras* L. J Fish Dis. 2011;34: 517–529.
3. Duesund H, Nylund S, Watanabe K, Ottem KF, Nylund A. Characterization of a VHS virus genotype III isolated from rainbow trout (*Oncorhychus mykiss*) at a marine site on the west coast of Norway. Virol J. 2010;7: 19.
4. Ito T, Kurita J, Sano M, Skall HF, Lorenzen N, Einer-Jensen K, et al. Typing of Viral Hemorrhagic Septicemia virus by monoclonal antibodies. J Gen Virol. 2012;93: 2546–2557.
5. Gagné N, MacKinnon AM, Boston L, Souter B, Cook-Versloot M, Griffiths S, et al. Isolation of Viral Haemorrhagic Septicemia virus from mummichog, stickleback, striped bass, and brown trout in eastern Canada. J Fish Dis. 2007;30: 213–223.
6. Einer-Jensen K, Ahrens P, Lorenzen N. Parallel phylogenetic analyses using the *N*, *G*, or *Nv* gene from a fixed group of VHSv isolates reveal the same overall genetic typing. Dis Aquat Organ. 2005;67: 39–45.
7. Basurco B, Benmansour A. Distant strains of the fish rhabdovirus VHSv maintain a sixth functional cistron, which codes for a nonstructural protein of unknown function. Virol. 1995;212(2): 741–745.
8. Benmansour A, Paubert G, Bernard J, de Kinkelin P. The polymerase-associated protein (*M1*) and the matrix protein (M*2*) from a virulent and an avirulent strain of Viral Hemorrhagic Septicemia virus (VHSv), a fish rhabdovirus. Virol. 1994;198(2): 602–612.
